# Implementing multi‐trait genomic selection to improve grain milling quality in oats (*Avena sativa* L.)

**DOI:** 10.1002/tpg2.20457

**Published:** 2024-05-19

**Authors:** Anup Dhakal, Jesse Poland, Laxman Adhikari, Ethan Faryna, Jason Fiedler, Jessica E. Rutkoski, Juan David Arbelaez

**Affiliations:** ^1^ Department of Crop Sciences University of Illinois Illinois Urbana USA; ^2^ Biological and Environmental Sciences and Engineering Division King Abdullah University of Science and Technology (KAUST), Thuwal, Saudi Arabia; ^3^ Center for Desert Agriculture, KAUST Thuwal Saudi Arabia; ^4^ Department of Plant Pathology Kansas State University Kansas Manhattan USA; ^5^ USDA–ARS Biosciences Research Laboratory Fargo North Dakota USA

## Abstract

Oats (*Avena* sativa L.) provide unique nutritional benefits and contribute to sustainable agricultural systems. Breeding high‐value oat varieties that meet milling industry standards is crucial for satisfying the demand for oat‐based food products. Test weight, thins, and groat percentage are primary traits that define oat milling quality and the final price of food‐grade oats. Conventional selection for milling quality is costly and burdensome. Multi‐trait genomic selection (MTGS) combines information from genome‐wide markers and secondary traits genetically correlated with primary traits to predict breeding values of primary traits on candidate breeding lines. MTGS can improve prediction accuracy and significantly accelerate the rate of genetic gain. In this study, we evaluated different MTGS models that used morphometric grain traits to improve prediction accuracy for primary grain quality traits within the constraints of a breeding program. We evaluated 558 breeding lines from the University of Illinois Oat Breeding Program across 2 years for primary milling traits, test weight, thins, and groat percentage, and secondary grain morphometric traits derived from kernel and groat images. Kernel morphometric traits were genetically correlated with test weight and thins percentage but were uncorrelated with groat percentage. For test weight and thins percentage, the MTGS model that included the kernel morphometric traits in both training and candidate sets outperformed single‐trait models by 52% and 59%, respectively. In contrast, MTGS models for groat percentage were not significantly better than the single‐trait model. We found that incorporating kernel morphometric traits can improve the genomic selection for test weight and thins percentage.

## Core Ideas

- Multi‐trait genomic selection improves the rate of genetic gain for milling traits in oat breeding programs.
- Kernel morphometric traits were more correlated with milling traits than groat morphometric traits.
- Phenotyping for highly correlated secondary traits in at least one single replication can improve the prediction accuracy for oat milling traits.

**Abbreviations**
GEBV: genomic estimated breeding value
GS: genomic selection
HTP: high‐throughput phenotyping
MTGS: multi‐trait genomic selection
STGS: single‐trait genomic selection

## 1 INTRODUCTION

Oats (*Avena sativa* L.) rank sixth among the most cultivated cereals worldwide (Mao et al., 2022). It is a multipurpose crop used in feed, fodder, and food (Marshall et al., 2013). Of the 25 million metric tons of oats harvested annually, only 14% are utilized in the food industry (Price & Welch, 2012). The demand for oats for human consumption, also known as food‐grade oats, is increasing because of oats’ distinctive nutritional and phytochemical characteristics (Martínez‐Villaluenga & Peñas, 2017; Rasane et al., 2015). Oats are a great source of dietary fiber, minerals, protein, fat, and bioactive compounds such as tocols, sterols, avenocosides, and avenanthramides (Butt et al., 2008; Michels et al., 2020). Oats’ soluble fibers, like beta‐glucan, can decrease the absorption of low‐density‐lipoprotein cholesterol, reducing the risk of cardiovascular diseases, the leading cause of death for people in the United States. (Grundy et al., 2017). Additionally, oats are a unique source of alternative gluten‐free products such as bread, beverages, granola bars, oat milk, cereals, biscuits, cookies, and porridge (Rasane et al., 2015; Tosh & Miller, 2015).

Food‐grade oats receive a premium price (Yan et al., 2013). Some mills pay up to $0.50–$0.60 more per bushel than feed‐grade oats (Brian Krause, personal communication, February 2024). For oats to be certified as food‐grade, they must fulfill different physical, chemical, and agronomical attributes. The requirement differs at each level of the value chain, but the revenue obtained by farmers is determined by grain milling industry standards (Esvelt Klos et al., 2021). Milling requirements vary depending on the production area and the individual mill needs (Girardet & Webster, 2011; USDA, 2020; Winkler & Murphy, 2017). Despite these differences, oat processors have common milling standards for food‐grade oats that include test weight values higher than 489 g/L, groat percentage higher than 72%, and thins percentage lower than 12% (Grain Millers, 2018; Joel et al., 2018). Failing to meet the thresholds set by millers for any of these quality attributes will result in discounts upon grain deliveries or complete seed lot dismissal (Grain Millers, 2018).

Test weight is defined as the weight of grains that fit a specific volume, expressed as lb/bu or g/L. Test weight is a rough indicator of grain quality. Test weight affects the cost of shipping and handling (Girardet & Webster, 2011). Groat percentage is calculated as the percentage of the weight of hull‐less kernels retained after dehulling a known amount of grain sample. Groat percentage is directly related to the milling yield (Groh et al., 2001). Thins percentage is the portion of the underdeveloped kernels that pass through the 5/64 × ¾‐inch screen relative to a known sample. Thin kernels are difficult to mill and require more energy for dehulling (Caffe et al., 2023), or are completely discarded during the milling process (Doehlert et al., 2001). A higher amount of thins indicates poor‐quality oats. These milling traits determine the efficiency of the milling process (Forsberg & Reeves, 2015) and drive the adoption and profitability of new oat varieties. Breeding for milling quality is fundamental for the future of the food‐grade oat industry.

In a breeding program, the evaluation for many milling attributes is delayed until later stages in the breeding pipeline. Evaluation of milling quality is labor‐intensive, requires many grains, and can be destructive (Aoun et al., 2022). For these reasons, breeding lines in their earlier stages are primarily evaluated and selected for agronomic and disease‐resistant traits. End‐use attributes like milling quality are delayed in their evaluation until advanced stages of phenotypic testing (Jernigan et al., 2018). Many times, advanced lines with excellent agronomic properties fail to meet the grain milling quality standards, making them unfit for variety release (Battenfield et al., 2016). To effectively improve grain milling quality in oats, we must evaluate and select breeding lines with superior milling quality as quickly as possible in the breeding pipeline. This can help us to rapidly identify parents with outstanding grain quality profiles, decreasing breeding cycle time and increasing the rate of genetic gain. For any trait, genetic gain is defined as the improvement of the average genetic value in a breeding population due to selection over breeding cycles (Hazel & Lush, 1942). Genetic gain is essential in a breeding program as it increases the probability of selecting superior individuals in each breeding cycle (Rutkoski, 2019). Higher rates of genetic gain ultimately improve variety development and adoption (Battenfield et al., 2016).

Genetic gain (∆G) can be predicted using the breeder's equation ∆G = (*σa*)(*i*)(*r*)/*L*, where *σa* is the additive genetic standard deviation within the population, *i* is the selection intensity, *r* is the selection accuracy, and *L* is the number of years per cycle (Falconer & Mackay, 1996). Selection accuracy is affected by the genetic architecture of the trait (Wientjes et al., 2022). Traits like test weight, groat percentage, and thins percentage, have complex genetic architecture controlled by many genes and environmental factors (De Koeyer et al., 2004; Esvelt Klos et al., 2021; Groh et al., 2001; Siripoonwiwat et al., 1996; Tanhuanpää et al., 2012).

Novel genomics and phenomics tools can help increase the rate of genetic gain for complex traits such as milling quality attributes (Merrick & Carter, 2021; Zhu et al., 2022). Genomic selection (GS) uses genome‐wide marker information to predict the performance of the candidate breeding lines that have not been phenotyped extensively or at all (Meuwissen et al., 2001). Marker effects are calculated in GS from the phenotypic and genotypic information available in a training population. Marker effects estimated using a GS model are then used to estimate the genomic estimated breeding value (GEBV) of the candidate set of lines (Robertsen et al., 2019). GS is advantageous for expensive and difficult‐to‐measure traits at earlier stages in the breeding pipeline. Selecting newly developed breeding lines based on GEBVs can substantially reduce the breeding cycle, consequently increasing the rate of genetic gain (Crossa et al., 2017; Jia & Jannink, 2012; Resende et al., 2012).

Phenomics is the systematic measurement and analysis of qualitative and quantitative traits of the plant (Arend et al., 2022). High‐throughput phenotyping (HTP) is a nondestructive and rapid platform to monitor and measure multiple traits related to anatomy, physiology, or biochemical properties (Pabuayon et al., 2019). HTP aims to measure traits of many breeding lines more accurately, precisely, and economically (Fritsche‐Neto et al., 2014; Sandhu et al., 2022). Secondary traits are those that are not economically important, but they are correlated with the economically important primary traits. HTP platforms can be used to measure secondary traits, and these traits could help improve the accuracy of GS when used in multi‐trait genomic selection (MTGS) models (Bhatta et al., 2020; Gaire et al., 2022; Rutkoski et al., 2016; Sandhu et al., 2021; Sun et al., 2017). With MTGS, the breeding value of milling quality traits can be estimated as soon as the lines are genotyped and phenotyped for HTP‐secondary traits (Bhatta et al., 2020; Sun et al., 2017). Simulated and real datasets have shown that MTGS has several advantages over single‐trait genomic selection (STGS) (Arojju et al., 2020; Atanda et al., 2022; Jia & Jannink, 2012).

Compared to other small grains, little research on integrating GS and HTP using MTGS for oat milling quality has been reported. Research on GS in oat has focused on training population design (Rio et al., 2021; Sørensen et al., 2023); STGS for seed metabolites (Campbell et al., 2021), glucan concentration (Asoro et al., 2013), and disease resistance (Haikka, Manninen et al., 2020); and MTGS for yield (Haikka, Knürr et al., 2020). In oats, grain morphometric features, including grain length, width, circularity, area, perimeter, and length‐to‐width ratio, are important for variety selection, as a certain type of grain shape and size is preferred by consumers and processors (Clohessy et al., 2018; Gustin & Settles, 2015). Seed morphometric traits can be measured using digital image analysis (Symons & Fulcher, 1988) and single‐seed analyzer system (Clohessy et al., 2018). In addition, instruments like Image S (Zimmer et al., 2021), 2D Scanner (Brzozowski et al., 2022), and Marvin Seed Analyzer (Howarth et al., 2021) are developed and used by breeding programs to measure the grain morphometric traits.

Previous studies have shown different degrees of genetic correlations between the oat grain morphometric features and different milling quality traits (Doehlert et al., 2006; Groh et al., 2001; Zimmer et al., 2021). Correlated grain morphometric traits can be used, along with genome‐wide molecular markers, in MTGS strategies to improve the prediction accuracy for test weight, groat, and thins percentage. Thus, the objectives of the study were to (i) implement HTP digital image analysis to extract secondary grain morphometric traits and estimate the degree of correlation with the milling quality traits across years in a diverse and large set of breeding lines, (ii) integrate HTP‐secondary morphometric grain traits in different MTGS strategies schemes and compare the models' prediction accuracy among each other and with STGS, and finally (iii) evaluate the advantage of MTGS strategies to reduce the number of replications to evaluate without comprising prediction accuracies.

**Core Ideas**

- Multi‐trait genomic selection improves the rate of genetic gain for milling traits in oat breeding programs.
- Kernel morphometric traits were more correlated with milling traits than groat morphometric traits.
- Phenotyping for highly correlated secondary traits in at least one single replication can improve the prediction accuracy for oat milling traits.

## 2 MATERIALS AND METHODS

### 2.1 Plant materials

Plant materials used in this study consisted of breeding lines from the University of Illinois at Urbana‐Champaign (UIUC) Spring Oat Breeding Program tested during the 2021 and 2022 breeding seasons. In the 2021 cohort, 403 breeding lines were planted in 1044 plots across four trial classes: preliminary, advanced, advanced‐early, and drill. In 2022, 356 breeding lines were tested in 912 plots across the same trial classes (Table S1). For this study, we chose 357 lines from 2021 and 336 lines from 2022. The chosen lines were hulled and had been genotyped previously. All trials were planted as randomized row‐column designs with three replicates in advanced, advanced‐early, and drill and two replicates for preliminary trials. Designs were generated using the R package DiGGer (Coombes, 2009). Four released varieties, Reins (Kolb et al., 2017), Excel, Spurs (Kolb et al., 2012), and Saber (Kolb et al., 2012), were planted as checks across all trials, and 147 lines overlapped across both years. Trials were planted at Urbana, IL, at UIUC's Crop Sciences Research and Education Center. Trials were planted using a six‐row Hege drill planter with plot sizes of 6 m^2^ or 4.5 m × 1.33 m, and a seed rate of 11.7 g/m^2^. Trials were fertilized with 40 lb/ac of nitrogen and sprayed with Callisto herbicide at a 3.0 fl. oz./ac rate.

### 2.2 Phenotyping

The workflow of this study is presented in Figure S1. Test weight was measured using the HarvestMaster H^3^ Classic Grain Gage Jupiner system mounted in a Winterstaiger's Classic Plus plot combine. Thins were measured using a 5/64 × ¾‐inch (1.98 mm × 19.05 mm) slotted screen mounted in Strand Sizer Sieve Shaker, Seedburo Equipment Company. Thins percentage is the percentage of the kernels passing through that slotted screen after 30 strokes (Esvelt Klos et al., 2021). A sample of 50 g of grain was collected from each plot and used to quantify thins and groat percentage. Groat percentage was measured by calculating the relative weight of groat from an original sample of 50 g of oat grain after dehulling using a Codema laboratory oat dehuller (Codema Inc.) (Esvelt Klos et al., 2021). Groat and thins percentage data were recorded using Field Book App (Rife & Poland, 2014). Phenotypic information on all the germplasm used in this research is publicly available on T3/Oat Breedbase (https://oat.triticeaetoolbox.org/) (Morales et al., 2022).

**FIGURE 1:**
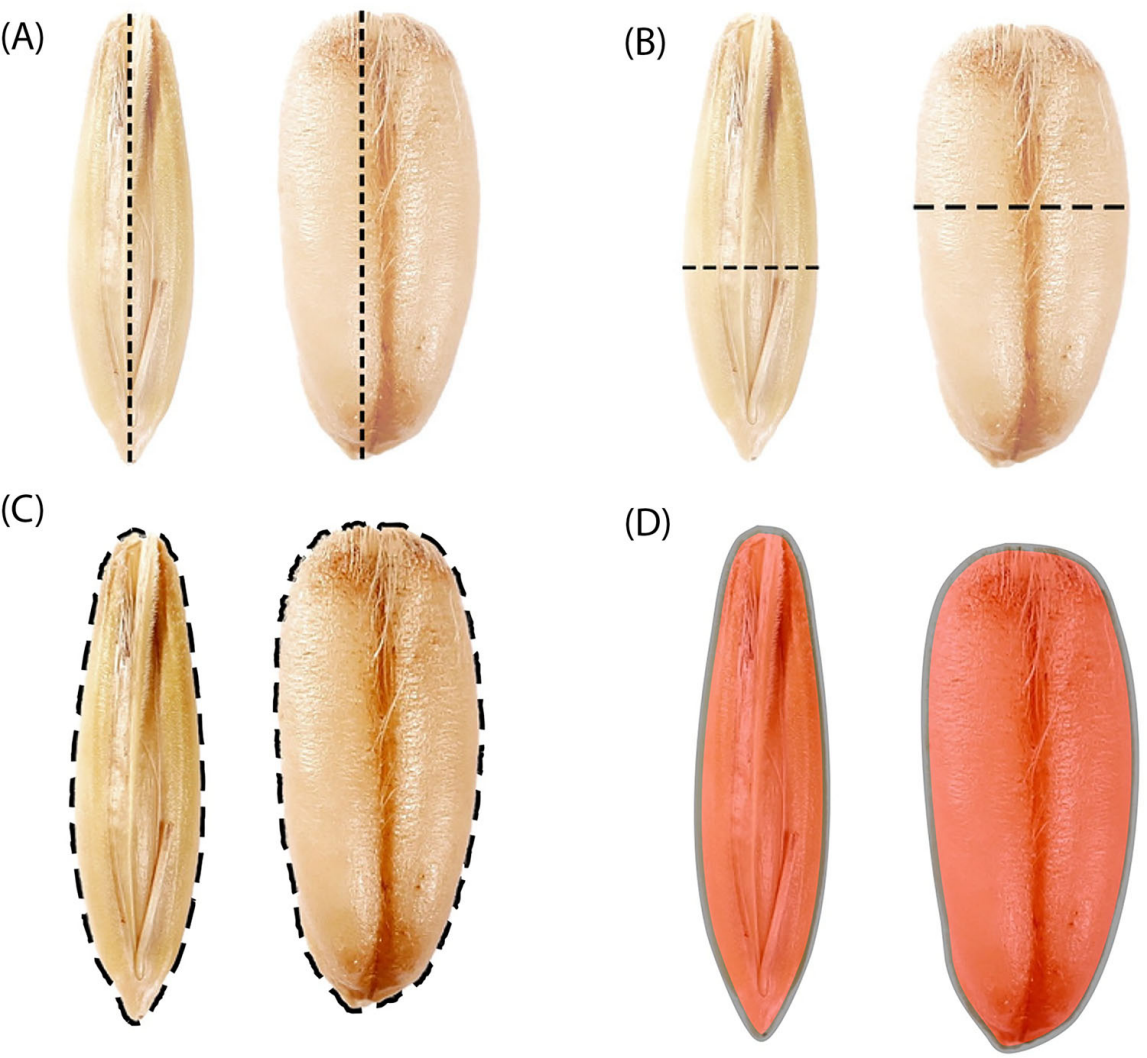
Different seed morphometric traits measured in kernel (left) and groat (right). (A) Major axis or length, (B) minor axis or width, (C) the dotted line measures the circumference or perimeter, and (D) red shade represents the area (Picture: modified from Adobe Stock #259622920).

For each sample, two images of ∼80 grains, as kernels (hulled grains) and groats (hulless grains), were taken using a mirrorless Nikon Z5 digital camera. Morphometric traits from kernels and groats images were estimated using the *Seed Extractor* package in MATLAB developed by Zhu et al. (2021). Four morphometric secondary traits, including length, width, perimeter, and area, were extracted and used for further analysis. The length or major axis is the length measured along the grove of the oat kernel or groat (Figure 1A), while the minor axis or width is the longest length across the perpendicular of the grove (Figure 1B). The perimeter is the length of the boundary of the seed or kernel (Figure 1C), and the area of the kernel or the groat is the number of pixels inside the seed or kernel region, respectively (Figure 1D).

Means and standard errors for each morphometric trait were estimated for each plot using 50 kernels or groats randomly selected as a representative sample of each plot. This number of kernels or groats was chosen based on previous analysis showing that 50 kernels or groats were sufficient to observe significant genetic differences between genotypes. These results were equivalent to those reported by (De Koeyer et al., 1993). In their study, they also recommended a similar number of seeds with high reproducibility and precision in grain morphometric traits evaluated.

### 2.3 Genotyping

Two different genotyping platforms were used to generate genome‐wide markers for all the breeding lines used in this study. The lines from the 2021 cohort were genotyped using genotyping‐by‐sequencing (GBS) (Elshire et al., 2011). 96‐plex libraries were prepared according to Poland et al. (2012). Single nucleotide polymorphisms (SNPs) were called using the TASSEL5 GBSv2 (Bradbury et al., 2007; Glaubitz et al., 2014) software. The sequencing reads were aligned to the *Avena sativa*—PepsiCo OT3098 v2 genome (PepsiCo, https://wheat.pw.usda.gov/jb?data=/ggds/oat-ot3098v2-pepsico) deposited in GrainGenes (Yao et al., 2022) using bowtie2 alignment (Langmead et al., 2009) and exported to variant call format (Langmead et al., 2009). The SNPs were then filtered for a minor allele frequency of 0.05, SNP called rate of 80%, and heterozygosity less than 5%. Missing SNP data were imputed using the LD KNNi imputation methodology with a high LD Sites 30 and number of nearest neighbors of 30 using LinkImpute algorithm (Money et al., 2015).

The 357 lines from the 2022 cohort and 397 lines from the 2021 cohort were genotyped using a genome‐wide Illumina Infinium XT genotyping array that enables the integration of custom multi‐species variants. The USDA‐SoyWheOatBar‐3k array contains informative SNPs for soybean, wheat, oat, and barley. For each crop, 3000 SNPs were built in the array. This array was designed by USDA‐ARS Small Grains Genotyping Laboratory, Fargo, ND, led by Dr. Jason Fiedler. Oat SNPs were called using Illumina's proprietary software Genome Studio (https://www.illumina.com).

Both genotyping sets were merged and imputed using Beagle 5.4 (Browning et al., 2018). The final genotyping data set can be found in T3/Oat (https://oat.triticeaetoolbox.org/). A final imputed set of 558 lines genotyped with 2149 markers was used for the following analyses. For all GS approaches, additive genomic relationship matrices (*K*) were estimated from the genotypic data using the *A.mat()* function in the rrBLUP package in R (Endelman, 2011).

### 2.4 Statistical analysis

#### 2.4.1 Calculation of best linear unbiased estimators and best linear unbiased predictor

GS models were carried out in two‐step approach. In the first step, adjusted means or best linear unbiased estimators (BLUEs) were calculated for each individual for each year to account for the trial variation and for spatial variation within trial. The mixed model was run using Asreml‐R version 4 (Butler et al., 2017) package in R (R Core Team, 2022).

The statistical model for step 1 was:

(1) $$y_{\mathrm{ijkl}}=\mu \;+\;G_{i}\;+\;T_{j}\;+\;GT_{ij}\;+\;R{\left(T\right)}_{ik}\;+\;C{\left(T\right)}_{il}+\varepsilon_{ijkl},$$

where $y_{ijkl}$ is the response variable for each trait for the *i*th genotype in *j*th trial place in *k*th row block and *l*th column block within the trial, μ is overall mean, $G_{i}$ is fixed effect of *i*th genotype (breeding lines), $\;T_{j}$ is fixed effect of *j*th trial, $G{\left(T\right)}_{ij}$ is random effect of *i*th germplasm in *j*th trial with normal distribution $∼N(0,\;\sigma_{GT}^{2})$, $R{\left(T\right)}_{ik}$ is random effect of *k*th row in *j*th trial with normal distribution $∼N(0,\;\sigma_{R}^{2})$, $C{\left(T\right)}_{il}$ is random effect of *i*th row in *j*th trial with normal distribution *∼* $N(0,\;\sigma_{C}^{2})$, and $\varepsilon_{ijkl}$ is residual random error term with normal distribution $∼N(0,\;\sigma_{E}^{2})$.

In addition to BLUEs, we also estimated the best linear unbiased predictor values (BLUPs). To calculate the BLUPs, we used the same model as described in Equation (1) but treated genotypes as a random effect instead of fixed.

Wald test (Wald, 1943) was used to determine if there was a significant difference among the genotype (breeding lines) and germplasm‐by‐year effect for all traits using *wald()* function in R (R Core Team, 2022).

#### 2.4.2 Heritability, genetic correlation, and principal component analysis

The broad sense heritability on entry mean basis of the traits was calculated using a similar model as Equation (1), where the genotype effect was treated as the random instead of a fixed effect. Broad sense heritability was calculated using the following equation (Hallauer et al., 2010):

(2) $$H^{2}=\frac{\sigma_{g}^{2}}{\sigma_{g}^{2}\;+\;\frac{\left(\sigma_{ge}^{2}\right)}{e}\;+\;\frac{\sigma_{e}^{2}}{e\;x\;r}\;}\;,$$

where $H^{2}$ is the broad sense heritability, $\sigma_{g}^{2}$ is the genotypic variance, $\sigma_{ge}^{2}$ is the genotype by environment interaction, $\sigma_{e}^{2}\;\mathrm{is}\;\mathrm{the}\;$ error variance, *e* is the mean number of trials where the genotype was tested, and *r* is the mean number of replications per genotype.

The genetic correlation among the traits was calculated using the unstructured genetic‐covariance matrices fitting a bivariate model (combination of all traits) using Asreml version 4 (Butler et al., 2017) in R (R Core Team, 2022) with a similar approach taken by Cruz et al. (2021).

(3) y=μ+Zg+e,

where ***y*** is the matrix of BLUEs of *n* genotype and two traits (*n* × 2), μ is mean vector of length (*n* × 2), ***Z*** is design matrix for random effect, ***g*** is vector of the random effect of genotype for both traits with g∼N(0,Σ⊗I), ***e*** is vector of residuals with e∼N(0,R⊗I), Σ and *R* are unstructured variance‐covariance matrices for the genetic and residual effect for each individual in both traits, ***I*** is the identity matrix, and ⊗ is Kronecker product of two matrices.

Principal component analysis was done using the “*factoextra*” package (Kassambara & Mundt, 2020) in R. All the graphs were created using the ggplot2 package (Wickham, 2016) in R (R Core Team, 2022).

#### 2.4.3 Genome wide association study

Genome‐wide association study (GWAS) was performed for primary milling and secondary grain morphometric traits using BLUPs by fitting the mixed linear model (Yu et al., 2006) at each marker using the gwas() function in rrBLUP package (Endelman, 2011) accounting for population structure and kinship. Based on the scree plot from the principal component analysis, the first three principal components were included in the model to account for population structure. The kinship matrix was calculated using the A.mat () function in rrBLUP package and used to account for familial relatedness. The study was carried out with an experiment‐wise false discovery rate of 5% using Bonferroni correction (Neyman & Pearson, 1928). As there were 2148 markers in the study with minor allele frequency >0.05, we fixed the Bonferroni correction value of *p* ≤ 2.327747 × 10^−5^. Manhattan plots and quantile–quantile (*Q*–*Q*) plots were generated using the qqman package (Turner, 2018) in R (R Core Team, 2022).

#### 2.4.4 Genomic selection

Single or MTGS models were performed using BLUEs estimated from Equation (1), and genome‐wide marker genetic information. The software ASReml‐R version 4 (Butler et al., 2017) in R (R Core Team, 2022) was used for the single and MTGS models. Each model performance was evaluated using a fivefold cross‐validation approach that was independently replicated five times. In the fivefold cross‐validation approach, genotypes or breeding lines were divided into five folds, with four folds used as the training set, and the remaining fold was masked and used as the candidate set.

##### Single‐trait genomic prediction

The ST‐GS used the following model:

(4) y=μ+Zg+e,

where ***y*** is vector of BLUEs for a single trait, **μ** is vector of overall mean for trait, ***Z*** is incidence matrix for the genotypic effect, ***g*** is vector of additive genotypic effect $∼N(0,\;K\sigma_{g}^{2})$, *r* is residual error vectors $\;∼N(0,\;I\;\sigma_{e}^{2})$, ***K*** is relationship matrix among the lines, $\sigma_{g}^{2}$ is additive genotypic variance, ***I*** is identity matrix, and $\sigma_{e}^{2}$ is error variance.

##### Multi‐trait genomic prediction

The following model was used in the MTGS:

(5) y=μ+Zg+e,

where **y** is a vector of BLUEs for traits (*n × t;*  *n* genotype and *t* traits), μ is the means of traits (vector of length *n × t)*, **Z** is the incidence matrix for the genomic effect of the lines, **g** is a vector of predicted genetic values of the individuals for all traits with

$$g∼\mathrm{MVN}\left(0,\Sigma ⊗K\right)$$

where **e** is a vector of residuals with

$$e∼\mathrm{MVN}\left(0,R⊗I\right)$$

where **K** is the realized additive relationship matrix among individuals estimated from the markers, ⊗ is Kronecker product of two matrices, *Σ* and *R* are the unstructured variance–covariance matrices for the genetic and residual effects between traits, respectively, and ***I*** is the identity matrix.

When marker information is masked, a similar model to Equation (5) is used with some modification. The BLUEs were obtained for each germplasm within a trial. The multi‐trait model fitted with the environment as a fixed effect, and interaction between the trait and germplasm was treated as random effect using an unstructured covariance matrix. The residual was assumed to be correlated for each combination of units and traits. The maximum number of iterations for fitting the model was increased to 20 to allow for convergence.

### 2.5 Cross‐validation scheme

**FIGURE 2:**
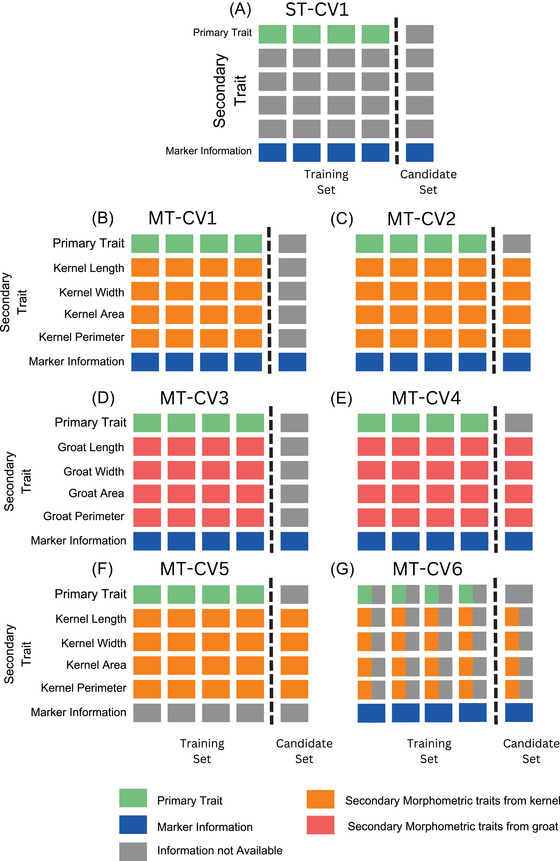
Cross‐validation schemes to test different models prediction accuracies for test weight, thins percentage, and groat percentage. (A) Single‐trait genomic prediction (ST‐CV1), (B) multi‐trait genomic prediction (MT) without secondary trait from the kernels in the candidate set (MT‐CV1), (C) MT with secondary traits from kernels (MT‐CV2), (D) MT without secondary trait from the groat in the candidate set (MT‐CV3), (E) MT with secondary trait from the groat in the candidate set (MT‐CV4), (F) MT with secondary traits from kernels but without marker information (MT‐CV5), and (G) MT with secondary trait from the kernels in the candidate set (MT‐CV2) but phenotypic information available is unreplicated (replication one only).

The reference cross‐validation scenario (ST‐CV1), evaluated the prediction accuracy of a single‐trait genomic selection (STGS) model when the training population had information on either primary trait, test weight, thins percentage, or groat percentage, and both training and candidate set had genotypic information (Figure 2A).

In the first multi‐trait cross‐validation scenario‐one (MT‐CV1), the training set had information on the primary trait, all secondary traits derived from the kernel images, and genotypic information. The candidate set had only genotypic information, missing primary and secondary trait information (Figure 2B). In the second multi‐trait cross‐validation scenario‐two (MT‐CV2), the training set had information on the primary trait, all secondary traits derived from the kernel images, and genotypic information. The candidate set had genotypic information and all secondary traits derived from the kernel images, but is missing the primary trait information (Figure 2C). The third multi‐trait cross‐validation scenario‐three scheme (MT‐CV3) (Figure 2D) was similar to MT‐CV1 but the secondary morphometric traits were measured from groat instead of kernel images. The fourth multi‐trait cross‐validation scheme (MT‐CV4) (Figure 2E) was similar to MT‐CV2 but in this scheme, secondary morphometric traits were measured from groat instead of kernel images. In the fifth multi‐trait cross‐validation scheme (MT‐CV5), training set had information on the primary trait, and all secondary traits derived from the kernel images. The candidate set had information in all secondary traits derived from the kernel images but is missing the primary trait information. Both training and candidate sets do not have genotypic information (Figure 2F). The final and sixth multi‐trait cross‐validation (MT‐CV6) scheme was similar to MT‐CV2, but in this case, the phenotypic information available is unreplicated. This last cross‐validation is how many programs evaluate grain quality attributes. In this scenario, grain samples are collected from one block from a typical replicated randomized complete block design (Figure 2G).

An additional multi‐trait cross‐validation scheme was designed to test if groat percentage prediction accuracies could be improved by including test weight as the only secondary trait (bivariate model). In this bivariate model scheme, the training set had information on groat percentage, test weight, and genotypic information. The candidate set had test weight and genotypic information.

## 3 RESULTS

### 3.1 Descriptive statistics, heritability, and genetic correlation

All traits, including test weight, thins percentage, groat percentage, grain length, width, circumference, and area, showed normal distributions across and within years (Figures S2, S3, and S4). Across years, test weight values ranged from 333 to 425 g/L, averaging 377 g/L. Thins percentage ranged from 4.6 to 36.8, with an average of 13, and groat percentage ranged from 61.4 to 72.3, with an average of 67.4. A summary of the phenotypic distributions and average values for primary and secondary traits can be found in Table S2. Our Wald test results showed that the genotypes were significant (*p*‐value < 0.001) in models where primary milling and secondary morphometric traits were used as dependent variables (Table S3). Wald test results also indicated that there was a significant genotype‐by‐year effect (*p*‐value < 0.001) for primary milling traits and groat morphometric traits.

**FIGURE 3:**
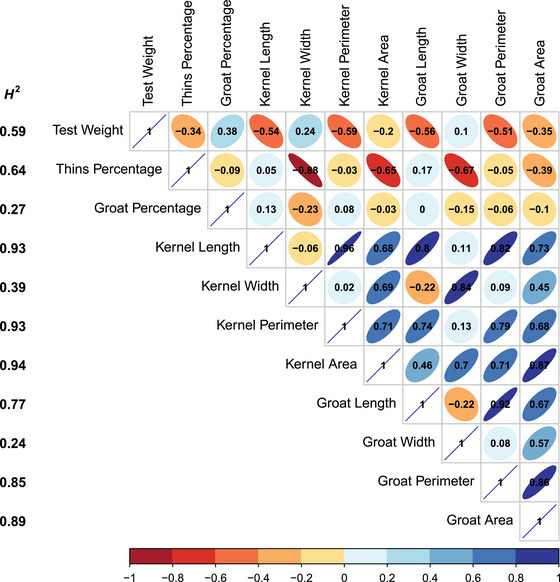
Broad sense heritability (*H* ^2^) in the vertical column and genetic correlation between the primary grain milling quality traits and secondary seed morphometric traits across years.

Broad sense heritability values for primary milling and secondary morphometric traits varied between them and years (Figure 3; Figures S5 and S6). In 2021, the broad sense heritability values for test weight, thins percentage, and groat percentage were 0.88, 0.87, and 0.88 (Figure S5). In 2022, heritability values for the same traits were 0.87, 0.86, and 0.87 (Figure S6). Across years, broad sense heritability values decreased to 0.59, 0.64, and 0.27 for test weight, thins percentage, and groat percentage, respectively (Figure 3).

We found weak and strong genetic correlations between primary milling quality and morphometric secondary traits (Figure 3). The direction of the correlations was consistent across years (Figure 3; Figures S5 and S6). However, the strength of the correlation changed across the years. Test weight was highly (*r* > 0.3) positively correlated with groat percentage and negatively correlated with thins percentage, kernel length, kernel perimeter, groat length, groat perimeter, and groat area (Figure 3). Thins percentage was highly negatively genetically correlated with test weight, kernel width, kernel area, groat width, and groat area. Groat percentage was only highly positively correlated with test weight (Figure 3). Most kernel and groat morphometric traits were positively correlated with each other except kernel width with groat length and groat length with groat width.

### 3.2 Genome‐wide association study

GWAS was performed using 558 genotypes and 2148 SNPs with an experiment‐wise false discovery rate of 5% for all primary and secondary traits. We did not find any significant association (*p* ≤ 2.327747 × 10^−5^) between any SNPs and all primary milling and secondary grain morphometric traits (Figure S7).

### 3.3 Single and multi‐trait genomic selection

**FIGURE 4:**
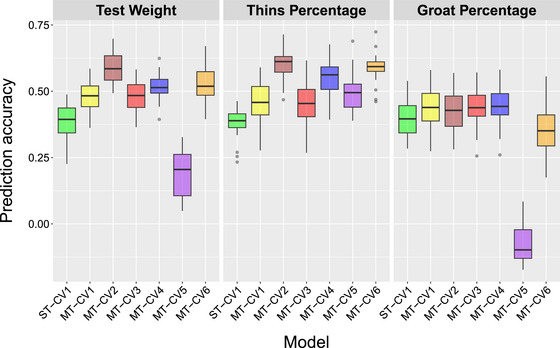
Box plot showing the prediction accuracy of different cross‐validation models for the three milling traits (test weight, thins percentage, and groat percentage) across both years.

All the models were compared with respect to the STGS (ST‐CV1), and the improvement of different MT models differed for each trait and year (Figure 4; Figures S8 and S9). The descriptive summary of the distribution of the prediction accuracy is presented in Table S4.

Across years, we observed that the mean prediction accuracy for test weight's ST‐CV1 model, or the single trait reference model, was 0.39. All multi‐trait models for test weight except MT‐CV5 performed better than ST‐CV1. From best to worst, MT‐CV2 (0.59), MT‐CV6 (0.53), MT‐CV4 (0.52), MT‐CV1 (0.48), and MT‐CV3 (0.48) outperformed ST‐CV1 (Figure 4). MT‐CV5, or the model where no molecular marker information was used, did not improve prediction accuracy compared to ST‐CV1. The models in which kernel secondary traits from training and candidate sets were included performed the best, that is, MT‐CV2 and MT‐CV6. The MT‐CV1 and MT‐CV3 models that did not include any secondary information on either kernel or groat traits in the candidate set were at the bottom.

All multi‐trait models performed better than the ST‐CV1 model for thins percentage. The mean prediction accuracy of ST‐CV1 was 0.38. The best‐ranked multi‐trait model was MT‐CV2 (0.6), followed by MT‐CV6 (0.59), MT‐CV4 (0.55), MT‐CV5 (0.49), MT‐CV1 (0.46), and finally MT‐CV‐3 (0.46). Just like test weight, the models including kernel secondary traits on training and candidate set performed the best, and the models with only kernel or groat secondary traits on training sets were at the bottom (Figure 4).

For groat percentage, all multi‐trait models except MT‐CV5 and MT‐CV6, performed slightly better than ST‐CV1. The multi‐trait models that outperformed ST‐CV1 only increase prediction accuracy by 0.03–0.05. MT‐CV6, the model with kernel secondary data obtained from unreplicated data, was less accurate than ST‐CV1 with a mean prediction accuracy of 0.35. MT‐CV5, the model without marker information, was unable to predict groat percentage at all (Figure 4).

The prediction accuracies within each year for test weight and thins percentage were consistent with those observed across years, with models ranking similarly (Figures S8 and S9). For groat percentage, we observed differences within each year (Figures S8 and S9). In 2021, four models, MT‐CV1, MT‐CV2, MT‐CV3, and MT‐CV4, performed slightly better than the univariate reference models ST‐CV1. However, in 2022 none of the multi‐trait models performed better than ST‐CV1.

## 4 DISCUSSION

Kernel and groat morphometric traits derived from digital images have been used to explain the genetic variation of oats grain milling attributes (De Koeyer et al., 1993; Doehlert et al., 1999; Pietrzak & Fulcher, 1995; Symons & Fulcher, 1988). Many of these studies were carried out using a limited amount of genotypes or diversity panels (Doehlert et al., 2004a; Symons & Fulcher, 1988; Zimmer et al., 2021). To determine the logistics, feasibility, and advantages of integrating GS and secondary grain morphometric traits to improve the prediction of grain milling quality at UIUC oat breeding program, we decided to use a large set of breeding lines that were yield tested in randomized replicated trials in 2021 and 2022.

Most oat programs sample one replicate from yield trials for evaluating milling quality. Though this approach eases logistics during harvesting and post harvesting, it makes estimation of heritability, repeatability, and genotype‐by‐environment less accurate. In our study, we sampled grains from replicated randomized trials and observed that repeatability expressed as broad sense heritability was high within each year for test weight, thins percentage, and groat percentage (*H* ^2^ > 0.86). Values for broad sense heritability for test weight, thins percentage, and groat percentage significantly decreased when estimated across years (*H* ^2^ < 0.64). These results implied a significant effect of genotype‐by‐year in the variance of our primary milling traits. We confirmed these observations by testing genotype‐by‐year effects for our primary traits using a Wald test (Table S3).

Previous linkage studies using biparental population or association mapping using diversity panels have identified some quantitative trait loci (QTLs)/markers associated with the milling traits and grain morphometric traits (De Koeyer et al., 2004; Esvelt Klos et al., 2021; Groh et al., 2001; Herrmann et al., 2014; Siripoonwiwat et al., 1996; Yan et al., 2023; Zimmer et al., 2021). Siripoonwiwat et al. (1996) using biparental inbred lines (Kanota × Ogle) found that 25 and 21 markers controlled test weight and groat percentage, respectively. Among these markers, the most significant four markers contributed 26% and 18% of the phenotypic variation. Groh et al. (2001), using the same recombinant inbred lines, found two QTLs explaining 22% of the total phenotypic variation for groat percentage and four QTLs explaining the 44.1% and 30.5% of the total phenotypic variation of kernel length and kernel width, respectively. However, these markers were significant only at a lower level of logarithm of odds score (2.5). Zimmer et al. (2021) used 406 lines in a diversity panel to map the genes for grain morphometric traits and found three markers for kernel length and one marker for kernel width. Still, each marker explained less than 5.5% of the total phenotypic variation. In a recent study, using the 501 lines of spring oat from the oat collaborative oat research enterprise panel evaluated in 13 locations, Esvelt Klos et al. (2021) found five mean QTLs for test weight, three mean QTLs for groat percentage, and one mean QTL for thins percentage across multiple year and locations. However, in our study, we did not see any significant association between the traits and markers. We used 558 lines from the breeding cohort of the UIUC oat breeding program and the major genes, if any, might have already been fixed by now. Yan et al. (2023), using 319 diverse lines in an oat panel, concluded that the major effect genes for grain morphometric traits were fixed in a majority of lines and minor effects alleles are yet to be fixed and could be utilized by the breeding program. Yan et al. (2023) also concluded that the additive nature of genes influencing grain morphometric traits. Considering that previous studies and our results showed that milling quality traits are complex with considerable genotype‐by‐year and environment interaction, and no major effect markers new strategies such as GS and secondary HTP traits can help us improve selection accuracy for test weight, thins percentage, and groat percentage.

In plant breeding, indirect selection for primary traits that are expensive or difficult to measure using highly heritable, correlated secondary traits is common. Our results showed that except for kernel width, all kernel morphometric traits had higher heritability values within and across years compared to the primary traits. We also observed no significant effect of genotype‐by‐year for kernel morphometric traits. In contrast, many groat morphometric traits had lower heritability values and had significant genotype‐by‐year effects compared to kernel traits.

Pleiotropic effects and linkage disequilibrium explain the level of shared genetic influence between two traits or their genetic correlation (Burdon, 2004; Hayes et al., 2017; Tsai et al., 2020). The strength in genetic correlation between multiple traits can be exploited in breeding for indirect selection or the effect on a trait due to selection on another trait (Roff, 1996). Oats’ test weight depends on the kernel density and packing efficiency (Doehlert et al., 2009). Seed shape and size are considered factors affecting test weight (Doehlert et al., 2004b; Forsberg & Reeves, 2015). Our results showed that test weight was negatively genetically correlated with the kernel length and kernel perimeter within and across the year. A similar relationship was also observed between the groat length and groat perimeter with test weight. Previous studies found negative correlations between test weight and kernel length (Doehlert et al., 2006; Forsberg & Reeves, 2015; Love, 1914). A longer kernel may contain air pockets beyond the groat, which lowers kernel density and test weight (Doehlert et al., 2006). Additionally, larger kernels have been shown to have poor packing efficiency (Doehlert et al., 2004b; Love, 1914). Although Love (1914) and Forsberg and Reeves (2015) concluded that the larger kernel width significantly improved packing efficiency, we observed a weak positive genetic correlation between test weight and kernel width (0.24).

Thins percentage was highly negatively correlated with kernel and groat width. This strong correlation might be due to the phenotyping process. Thins are calculated based on the weight of the undersized kernel that passed through the 1.98 mm sieve (Girardet & Webster, 2011). These sieves are usually 19.05‐mm long by 1.98‐mm wide (Doehlert et al., 2004b). The sieve's length is larger than the normal kernel length. Thus, there was no significant correlation between the thins percentage and kernel length.

There is no significant genetic correlation between any seed morphometric trait and groat percentage, except for a weak correlation with kernel width. Past research does not show a consistent relationship between kernel or groat morphology and groat percentage. A previous study that evaluated 10 cultivars across five locations found a high correlation of groat percentage with groat area, length, and width (Doehlert et al., 2006). Another study that evaluated 10 oat genotypes across three environments showed no significant correlation between the groat percentage and kernel morphometric traits (Doehlert et al., 2009). This inconsistent relationship between the groat and kernel morphometric traits may be related to different genetic backgrounds and environments (Groh et al., 2001). Our UIUC breeding material had a poor genetic correlation between groat percentage, kernel, and groat morphometric traits.

Although no significant correlation was observed between the primary traits test weight, thins percentage, and groat percentage in 2021, a moderate genetic correlation between test weight and thins percentage and groat percentage was found in 2022. The negative genetic correlation between test weight and thins indicates that undeveloped and undersized kernels negatively impact the test weight. A large germplasm set of 501 spring oats showed a similar negative relationship (Esvelt Klos et al., 2021). The positive relationship between the test weight and groat percentage has been reported in previous research (Doehlert et al., 2009; Souza & Sorrells, 1988). The positive genetic correlation is favorable as selection for one trait might increase the value for another trait.

Overall, we observed that kernel morphometric traits have high heritabilities and have higher genetic correlations with test weight and thins percentage as compared to groat morphometric traits. No secondary traits in our study were significantly correlated with groat percentage. Kernel morphometric traits are ideal to be included in MTGS strategies for test weight and thins percentage.

To better understand how GS can benefit from including secondary traits, we incorporate the information from the kernel and groat morphometric traits into MTGS models to improve prediction accuracy compared to single trait models. This study also aims to study the advantage of incorporating genetic information in the multi‐trait model. We also studied how phenotyping can be optimized by reducing the number of replications for phenotyping.

The improvement of prediction accuracy compared to a single trait (ST) varied for different cross‐validation schemes, traits, years, and sets of secondary traits (Figure 4; Figures S6 and S7). The result indicates a minor or slight improvement in the prediction accuracy of test weight and thins percentage even when secondary information on kernel or groat morphometric traits was unavailable on candidate lines. The slight improvement in prediction accuracy of those traits is due to the inclusion of highly heritable and strongly correlated secondary traits (Guo et al., 2014; Jia & Jannink, 2012; Okeke et al., 2017). However, there was no additional advantage when predicting the new and untested lines for groat percentage when information on secondary traits was included only in the training set (MT‐CV1 vs. ST‐CV1 and MT‐CV3 vs. ST‐CV1). Similar results were obtained in previous studies where no information was available for secondary traits in multi‐trait models (Bhatta et al., 2020; Gaire et al., 2022; Lado et al., 2018; Shahi et al., 2022). Lado et al. (2018) and Arojju et al. (2020) suggested that a small training population might limit the benefit of MT‐CV1; however, we did not see that limitation for test weight and thins percentage. MT‐CV1 and MT‐CV3 are more computationally demanding than ST, and such minimal improvement over ST can be an actual restriction for regularly using MT‐CV1 and MT‐CV3 over ST.

In MT‐CV2 and MT‐CV4 models, training and candidate lines have additional information on secondary morphometric traits from kernel and groat, respectively. That additional information on correlated secondary traits increases the prediction accuracy (Okeke et al., 2017; Rutkoski et al., 2016; Sun et al., 2017; Winn et al., 2023). Similar to these previous studies, MT‐CV2 performed better to the respective ST‐CV1, and MT‐CV4 performed better than the respective ST‐CV1 and MT‐CV3 for all traits. For test weight and thins percentage, MT‐CV2 was the best model, while MT‐CV2 and MT‐CV4 performed similarly for groat percentage. The extent of the improvement of MT‐CV2 or MT‐CV4 over ST‐CV1 varied for traits despite using the same secondary trait, which may be due to the variation in genetic correlation among the primary and secondary traits and heritability of the primary trait. However, to measure the seed morphometric traits in groat, the kernels must be manually dehulled or using the machine. Besides the time‐consuming process, dehulling might also damage the groat, which makes the groat unsuitable to be used as the seed source. Thus, using data from groat images is unjustified. We also did not include any model incorporating both kernel and groat morphometric traits, as these traits were highly colinear or correlated. Multi‐trait models with colinear traits create problems with model convergence (Okeke et al., 2017), and adding secondary traits to a multi‐trait model that are highly correlated with other traits in the model has little to no advantage (Schulthess et al., 2016).

Multi‐trait models can predict the breeding value without marker information solely based on phenotypic information on multiple traits (Henderson & Quaas, 1976). We wanted to test how well secondary kernel morphometric traits predict the milling quality without the marker information using MT‐CV5 scheme. Although this model predicted thins percentage better than the ST, it performed worse than ST for test weight, and could not predict groat percentage at all. Kernel morphometric traits (kernel width and kernel area) were highly correlated (*r* > 0.6) with thins percentage, but test weight and groat percentage lacked such a strong correlation. When highly correlated traits were available, Lado et al. (2018) found marginal benefits of including marker information. In cases like ours, where the correlation between secondary and primary traits is low, marker information becomes important in MT to obtain a higher prediction accuracy. Calus and Veerkamp (2011) and Fernandes et al. (2018) suggest that when the secondary trait had a genetic correlation smaller than 0.5, secondary traits might not benefit the multi‐trait model without marker information, and thus, genotypic information becomes crucial in such conditions. Although it is possible to increase the prediction accuracy with a large training population when the secondary trait has a low genetic correlation (Fernandes et al., 2018), in our case, the training size was not enough to enhance the prediction accuracy for groat percentage. It is unclear how large the training population would need to be to overcome the effect of low genetic correlation, as other factors like heritability are also involved (Calus & Veerkamp, 2011).

Time and resources are limited in a breeding program, but numerous lines must be phenotyped yearly. Phenotyping is a key bottleneck in plant breeding (Araus et al., 2018; Crain et al., 2022). Studies on different crops were carried out to optimize the resource using MTGS. Some of those optimizations include reduced phenotyping (50% of newer lines) (Gaire et al., 2022; Lado et al., 2018), sparse testing in multiple environments (Jarquin et al., 2020; Montesinos‐López et al., 2021), and phenotyping subset of highly correlated traits (Gaire et al., 2022). Extensive phenotyping on replicated trials is not feasible for early generations (Krause et al., 2020), or sometimes just one replication is phenotyped for selection due to logistic management during harvesting and post‐harvesting. Thus, we wanted to evaluate if the information from a single replication can optimize the phenotyping resource. For test weight and thins percentage, MT‐CV6 performed better than ST‐CV1, indicating that including information on secondary traits from one replication in MTGS significantly increases the prediction accuracy. This might be due to the high heritability of those secondary traits. However, for groat percentage, the model with kernel secondary data obtained from unreplicated data was less accurate than ST‐CV1. MT‐CV6 was superior to MT‐CV1 but the advantage was limited compared to MT‐CV2 for both test weight and thins percentage. This indicates that under a resource constraint scenario or for easy logistics, it is better to include information on secondary traits from one replication (unreplicated) design rather than completely excluding them from the model. MT‐CV6 can be vital in early generations such as first‐year yield trials or research designs that are impossible to replicate. MT‐CV6 represents a resource‐constrained situation and indicates that if we have limited resources, we can still leverage prediction efficiency by phenotyping a single replication.

The prediction accuracy of the multi‐trait model depends on numerous factors like genetic architecture of trait, relatedness between training and prediction population, heritability of the secondary trait, the statistical model used, and genetic correlation between the primary and secondary trait (Fernandes et al., 2018; Gevartosky et al., 2023; Lozada & Carter, 2019; Muvunyi et al., 2022). Each factor affects the prediction accuracy, but there are limited studies on how the combination of those factors works (Atanda et al., 2022). In many cases, individual factors have been explained independently. In this research, three primary traits have different genetic architectures (Esvelt Klos et al., 2021), which might be one reason for the variation in the prediction accuracy across different traits despite using the same set of secondary traits. We did not see any population structure (Figure S10) among the germplasm as all the lines were from the UIUC oat breeding program, so there is a minimum effect of the population structure on the prediction accuracy. We believe the most important factor determining prediction accuracy in this study was genetic correlation between the primary and secondary traits. The correlation of the traits is so important that when the secondary traits are highly correlated, the size of the training population can be reduced. Previous research found that training population size can be reduced by 30%–50% to maintain a similar prediction accuracy when highly correlated traits were used as secondary traits (Arojju et al., 2020; Lado et al., 2018). Furthermore, a strong genetic correlation between the primary and secondary traits can improve the prediction accuracy despite the low heritability of the secondary trait (Muvunyi et al., 2022). Generally, less heritable traits like groat percentage should have performed better under MT‐GS when the highly heritable secondary traits are included in the model. However, this was not the case in this study, and genetic correlation might be the limiting factor.

MTGS is beneficial when evaluating the second trait and is cost‐effective, easier, and earlier than measuring the target trait directly (Schulthess et al., 2016). As the training population is updated regularly, increasing the training population size, MTGS is expected to result in higher prediction accuracy (Montesinos‐López et al., 2021). In our case, HTP platforms can be used to measure kernel morphometric traits as early as the first‐year yield trial, and MTGS can be implemented. However, using only kernel morphometric traits for groat percentage without marker information is not recommended. Genotypic information is very crucial for the estimation of groat percentage, and MTGS or GS can be implemented as soon as genotyping is done. An earlier selection of breeding candidates using MTGS will also reduce the breeding cycle, thus increasing the genetic gain for the milling traits (Figure S11). The success of the GS in a breeding program is not only determined by the prediction accuracy. How breeders use the information from GS, HTP, and phenotypic evaluation to advance lines, or parental selection is essential for the success of the breeding strategy (Lozada & Carter, 2019).

In this study, we used the same secondary morphometric traits for all milling quality traits. But, we would need to consider using a different set of secondary traits for different milling traits, especially groat percentage. Other traits that are easy to phenotype and genetically correlated with groat percentage must be identified. For this, we can evaluate other traits like 100‐kernel weight or mass:area ratio, as these were correlated to groat percentage with a smaller germplasm set (Doehlert et al., 1999). Test weight is another factor that is highly related to the groat percentage (Doehlert et al., 1999), and test weight can be used as a predictor trait in a multi‐trait model, provided information on test weight is available. There are harvesters (e.g., HarvestMaster's Classic GrainGage) with technology that measures yield, moisture, and test weight during the harvesting. To stimulate a similar scenario, we ran a bivariate model with groat percentage and thins percentage as a primary and test weight as a predictor trait. We found out that the prediction accuracy increased by 27.17%–31.47% compared to a STGS (Figure S12). This increase in the predictive accuracy was higher than any MT model for groat percentage with secondary morphometric traits. Breeding programs with such technology where phenotypic information on test weight is readily available, can benefit from additional information from test weight instead of seed morphometric traits for the groat percentage.

This study was based on a single location across 2 years. Thus, further study with multiple environments and years is required to see how MTGS becomes effective in such a situation (Atanda et al., 2022).

## 5 SUMMARY

Oats must fulfill strict industry grain milling quality standards for food use. Phenotyping for milling quality traits is delayed until the later stages of breeding. Most advanced selection candidates may fail to meet those milling requirements despite having good agronomics. Grain morphometric traits, for example, length and width, can be phenotyped earlier in the breeding pipeline at a fraction of the cost. Including morphometric traits in genomic prediction models can improve their performance and allow breeders to make selections faster and cheaper.

## AUTHOR CONTRIBUTIONS

**Anup Dhakal**: Data curation; formal analysis; investigation; methodology; visualization; writing—original draft. **Jesse Poland**: Methodology; resources; software. **Laxman Adhikari**: Data curation; methodology; resources; software. **Ethan Faryna**: Data curation; methodology; resources. **Jason Fiedler**: Data curation; methodology; resources; software. **Jessica E. Rutkoski**: Formal analysis; methodology; writing—review and editing. **Juan David Arbelaez**: Conceptualization; formal analysis; funding acquisition; writing—review and editing.

## CONFLICT OF INTEREST STATEMENT

The authors declare no conflicts of interest.

## Supporting information

Supplementary Figure 1: Workflow of the study.
Supplementary Figure 2: Distribution of the primary and secondary traits in year 2021. Supplementary Figure 3: Distribution of the primary and secondary traits in year 2022.
Supplementary Figure 4: Distribution of the primary and secondary traits across the year 2021 and 2022.
Supplementary Figure 5: Broad sense heritability value and genetic correlation between the primary milling and secondary morphometric traits in 2021.
Supplementary Figure 6: Broad sense heritability value and genetic correlation between the primary milling and secondary morphometric traits in 2022.
Supplementary Figure 7: Manhattan plot and QQplot for GWAS result of primary milling traits.
Supplementary Figure 8: Prediction accuracy of different genomic selection cross‐validation scheme for milling traits in 2021.
Supplementary Figure 9: Prediction accuracy of different genomic selection cross‐validation scheme for milling traits in 2022.
Supplementary Figure 10: Population structure among the germplasm used in this study.
Supplementary Figure 11: A typical and proposed breeding pipeline for breeding for milling traits in oat.
Supplementary Figure 12: Prediction accuracy of Bivariate model predicting thins percentage and groat percentage using test weight as predictor traits.

Supplementary Table 1: List of accessions and trials where those accessions were used.
Supplementary Table 2: Descriptive summary of BLUPs of traits measured in 2021, 2022 and combined across the years.
Supplementary Table 3: Wald test result of primary and secondary traits across the years.
Supplementary Table 4: Descriptive summary of prediction accuracy of different genomic selection cross‐validation schemes.

## Data Availability

Phenotypic information used in this study is available at T3/Oat database at https://oat.triticeaetoolbox.org/.
