# Supplementary material for: Implementing multi‐trait genomic selection to improve grain milling quality in oats (Avena sativa L.)
Source: Plant Genome. 2024 May 19;17(2):e20457. doi: 10.1002/tpg2.20457 (PMC12807195; doi:10.1002/tpg2.20457)
Supplement: Supplementary file 1 [file TPG2-17-e20457-s001.docx]

**SUPPLEMENTARY TABLES**

**Implementing multi-trait genomic selection to improve grain milling quality in oats (*Avena sativa* L.)**

**Anup Dhakal, Jesse Poland, Laxman Adhikari, Ethan Faryna, Jason Fiedler, Jessica E. Rutkoski, and Juan David Arbelaez.**

Supplementary Table 1: List of accessions and number of trials those accessions were used

Abbreviation:

Adv: Advanced Yield Trial

AE: Advanced Early Yield Trial

Drl: Drill Yield Trial

Pr: Preliminary Yield Trial

Urb: Urbana

21: Year 2021

22: Year 2022

| Accession | Experiment | Number of trials |
| --- | --- | --- |
| BAKER | Drl_Urb_21, Drl_Urb_22 | 2 |
| BUCKSKIN | Drl_Urb_21, Adv_Urb_22, AE_Urb_22, Drl_Urb_22 | 4 |
| COLT | Drl_Urb_21, Drl_Urb_22 | 2 |
| CORRAL | Adv_Urb_22 | 1 |
| DEON | Drl_Urb_21, Drl_Urb_22 | 2 |
| ESKER | Drl_Urb_21, Drl_Urb_22 | 2 |
| EXCEL | Adv_Urb_21, AE_Urb_21, Drl_Urb_21, Pr_Urb_21, Adv_Urb_22, AE_Urb_22, Drl_Urb_22 | 7 |
| HAYDEN | Drl_Urb_21, Drl_Urb_22 | 2 |
| HORSEPOWER | Drl_Urb_21, Drl_Urb_22 | 2 |
| IL10-9872 | AE_Urb_21, Drl_Urb_21 | 2 |
| IL12-6842 | AE_Urb_21, Drl_Urb_21 | 2 |
| IL14-8142 | Adv_Urb_21, Drl_Urb_21, Drl_Urb_22 | 3 |
| IL14-9617 | Adv_Urb_21, Drl_Urb_22 | 2 |
| IL15-2064 | AE_Urb_21, Drl_Urb_21, Drl_Urb_22 | 3 |
| IL15-2368 | Adv_Urb_21, Drl_Urb_21, Drl_Urb_22 | 3 |
| IL15-3316 | Adv_Urb_21, Drl_Urb_21, Drl_Urb_22 | 3 |
| IL15-5271 | Adv_Urb_21, Drl_Urb_22 | 2 |
| IL15-5312 | Adv_Urb_21 | 1 |
| IL15-5752 | AE_Urb_21, Drl_Urb_21, Drl_Urb_22 | 3 |
| IL15-5760 | AE_Urb_21, Drl_Urb_21, Drl_Urb_22 | 3 |
| IL16-11067 | AE_Urb_21, Drl_Urb_21 | 2 |
| IL16-11441 | Drl_Urb_21, Drl_Urb_22 | 2 |
| IL16-6760 | AE_Urb_21, Drl_Urb_21 | 2 |
| IL16-9239 | Drl_Urb_21, Drl_Urb_22 | 2 |
| IL17-10306 | Adv_Urb_21, Drl_Urb_21, Drl_Urb_22 | 3 |
| IL17-11228 | Adv_Urb_21, Drl_Urb_21, Drl_Urb_22 | 3 |
| IL17-11726 | Drl_Urb_21 | 1 |
| IL17-12162 | Adv_Urb_21 | 1 |
| IL17-1253 | Adv_Urb_21, Drl_Urb_22 | 2 |
| IL17-1333 | Adv_Urb_21, Drl_Urb_21, Drl_Urb_22 | 3 |
| IL17-1677 | Drl_Urb_21, Drl_Urb_22 | 2 |
| IL17-1704 | Adv_Urb_21, Drl_Urb_22 | 2 |
| IL17-2965 | Adv_Urb_21, Drl_Urb_21, Drl_Urb_22 | 3 |
| IL17-3125 | AE_Urb_21, Drl_Urb_21, Drl_Urb_22 | 3 |
| IL17-3503 | Adv_Urb_21 | 1 |
| IL17-3920 | AE_Urb_21, Drl_Urb_21, Drl_Urb_22 | 3 |
| IL17-4927 | AE_Urb_21, Drl_Urb_21, Drl_Urb_22 | 3 |
| IL17-5238 | AE_Urb_21, Drl_Urb_21 | 2 |
| IL17-7278 | Adv_Urb_21, Drl_Urb_21 | 2 |
| IL17-7334 | Adv_Urb_21, Drl_Urb_22 | 2 |
| IL17-7339 | Adv_Urb_21, Drl_Urb_21, Drl_Urb_22 | 3 |
| IL17-8278 | Adv_Urb_21, Drl_Urb_21, Drl_Urb_22 | 3 |
| IL17-8857 | AE_Urb_21, Drl_Urb_21, Drl_Urb_22 | 3 |
| IL18-1064 | Adv_Urb_21, Drl_Urb_22 | 2 |
| IL18-1107 | Adv_Urb_21, Drl_Urb_22 | 2 |
| IL18-1148 | AE_Urb_21 | 1 |
| IL18-1568 | Adv_Urb_21 | 1 |
| IL18-2308 | Adv_Urb_21, Drl_Urb_22 | 2 |
| IL18-2328 | Adv_Urb_21 | 1 |
| IL18-2334 | Adv_Urb_21 | 1 |
| IL18-2519 | AE_Urb_21 | 1 |
| IL18-2658 | AE_Urb_21 | 1 |
| IL18-2856 | AE_Urb_21 | 1 |
| IL18-2908 | AE_Urb_21 | 1 |
| IL18-2936 | AE_Urb_21 | 1 |
| IL18-2945 | Adv_Urb_21, Drl_Urb_22 | 2 |
| IL18-3344 | AE_Urb_21 | 1 |
| IL18-3375 | AE_Urb_21 | 1 |
| IL18-3639 | AE_Urb_21, Drl_Urb_22 | 2 |
| IL18-4344 | AE_Urb_21 | 1 |
| IL18-4379 | Adv_Urb_21 | 1 |
| IL18-4515 | AE_Urb_21, Drl_Urb_22 | 2 |
| IL18-4643 | AE_Urb_21 | 1 |
| IL18-4663 | Adv_Urb_21, Drl_Urb_22 | 2 |
| IL18-4707 | Adv_Urb_21 | 1 |
| IL18-5231 | Adv_Urb_21 | 1 |
| IL18-5248 | Adv_Urb_21 | 1 |
| IL18-5250 | Adv_Urb_21 | 1 |
| IL18-574 | AE_Urb_21 | 1 |
| IL18-5968 | Adv_Urb_21 | 1 |
| IL18-6323 | Adv_Urb_21, Drl_Urb_22 | 2 |
| IL18-6368 | Adv_Urb_21 | 1 |
| IL18-6972 | AE_Urb_21 | 1 |
| IL18-735 | Adv_Urb_21, Drl_Urb_22 | 2 |
| IL18-7630 | Adv_Urb_21 | 1 |
| IL18-7913 | Adv_Urb_21 | 1 |
| IL18-805 | AE_Urb_21, Drl_Urb_22 | 2 |
| IL18-8244 | Adv_Urb_21, Drl_Urb_22 | 2 |
| IL18-827 | AE_Urb_21, Drl_Urb_22 | 2 |
| IL18-8479 | AE_Urb_21 | 1 |
| IL18-8562 | Adv_Urb_21, Drl_Urb_22 | 2 |
| IL18-8722 | AE_Urb_21, Drl_Urb_22 | 2 |
| IL18-9105 | AE_Urb_21, Drl_Urb_22 | 2 |
| IL19-110 | Pr_Urb_21 | 1 |
| IL19-1111 | Pr_Urb_21 | 1 |
| IL19-1161 | Pr_Urb_21 | 1 |
| IL19-1222 | Pr_Urb_21, Adv_Urb_22 | 2 |
| IL19-1234 | Pr_Urb_21 | 1 |
| IL19-1235 | Pr_Urb_21, AE_Urb_22 | 2 |
| IL19-1241 | Pr_Urb_21 | 1 |
| IL19-127 | Pr_Urb_21 | 1 |
| IL19-1365 | Pr_Urb_21 | 1 |
| IL19-1370 | Pr_Urb_21 | 1 |
| IL19-1402 | Pr_Urb_21, Adv_Urb_22 | 2 |
| IL19-1454 | Pr_Urb_21 | 1 |
| IL19-1459 | Pr_Urb_21 | 1 |
| IL19-1472 | Pr_Urb_21 | 1 |
| IL19-1474 | Pr_Urb_21 | 1 |
| IL19-1509 | Pr_Urb_21 | 1 |
| IL19-1515 | Pr_Urb_21 | 1 |
| IL19-1518 | Pr_Urb_21, Adv_Urb_22 | 2 |
| IL19-1524 | Pr_Urb_21 | 1 |
| IL19-1525 | Pr_Urb_21, Adv_Urb_22 | 2 |
| IL19-1526 | Pr_Urb_21 | 1 |
| IL19-1558 | Pr_Urb_21, Adv_Urb_22 | 2 |
| IL19-1574 | Pr_Urb_21 | 1 |
| IL19-1670 | Pr_Urb_21 | 1 |
| IL19-1702 | Pr_Urb_21 | 1 |
| IL19-1811 | Pr_Urb_21, Adv_Urb_22 | 2 |
| IL19-1819 | Pr_Urb_21 | 1 |
| IL19-1823 | Pr_Urb_21, Adv_Urb_22 | 2 |
| IL19-1873 | Pr_Urb_21 | 1 |
| IL19-1971 | Pr_Urb_21 | 1 |
| IL19-1974 | Pr_Urb_21 | 1 |
| IL19-2016 | Pr_Urb_21 | 1 |
| IL19-2033 | Pr_Urb_21, Adv_Urb_22 | 2 |
| IL19-2049 | Pr_Urb_21 | 1 |
| IL19-2053 | Pr_Urb_21 | 1 |
| IL19-2057 | Pr_Urb_21 | 1 |
| IL19-2060 | Pr_Urb_21 | 1 |
| IL19-2073 | Pr_Urb_21 | 1 |
| IL19-2078 | Pr_Urb_21, Adv_Urb_22 | 2 |
| IL19-2129 | Pr_Urb_21 | 1 |
| IL19-213 | Pr_Urb_21, Adv_Urb_22 | 2 |
| IL19-2133 | Pr_Urb_21 | 1 |
| IL19-2150 | Pr_Urb_21 | 1 |
| IL19-2155 | Pr_Urb_21, Adv_Urb_22 | 2 |
| IL19-216 | Pr_Urb_21 | 1 |
| IL19-2213 | Pr_Urb_21, AE_Urb_22 | 2 |
| IL19-2245 | Pr_Urb_21, Adv_Urb_22 | 2 |
| IL19-232 | Pr_Urb_21 | 1 |
| IL19-2348 | Pr_Urb_21 | 1 |
| IL19-2467 | Pr_Urb_21 | 1 |
| IL19-2501 | Pr_Urb_21 | 1 |
| IL19-2512 | Pr_Urb_21 | 1 |
| IL19-2576 | Pr_Urb_21 | 1 |
| IL19-2606 | Pr_Urb_21, Adv_Urb_22 | 2 |
| IL19-2608 | Pr_Urb_21 | 1 |
| IL19-2628 | Pr_Urb_21 | 1 |
| IL19-2629 | Pr_Urb_21, Adv_Urb_22 | 2 |
| IL19-2630 | Pr_Urb_21, Adv_Urb_22 | 2 |
| IL19-2655 | Pr_Urb_21, AE_Urb_22 | 2 |
| IL19-2673 | Pr_Urb_21, AE_Urb_22 | 2 |
| IL19-2710 | Pr_Urb_21 | 1 |
| IL19-2728 | Pr_Urb_21 | 1 |
| IL19-2730 | Pr_Urb_21 | 1 |
| IL19-2766 | Pr_Urb_21 | 1 |
| IL19-2769 | Pr_Urb_21 | 1 |
| IL19-2770 | Pr_Urb_21, AE_Urb_22 | 2 |
| IL19-2843 | Pr_Urb_21, Adv_Urb_22 | 2 |
| IL19-3011 | Pr_Urb_21 | 1 |
| IL19-3015 | Pr_Urb_21, Adv_Urb_22 | 2 |
| IL19-3023 | Pr_Urb_21 | 1 |
| IL19-3025 | Pr_Urb_21 | 1 |
| IL19-3038 | Pr_Urb_21 | 1 |
| IL19-3039 | Pr_Urb_21 | 1 |
| IL19-3041 | Pr_Urb_21 | 1 |
| IL19-3067 | Pr_Urb_21 | 1 |
| IL19-3068 | Pr_Urb_21, AE_Urb_22 | 2 |
| IL19-3069 | Pr_Urb_21 | 1 |
| IL19-3104 | Pr_Urb_21 | 1 |
| IL19-3146 | Pr_Urb_21 | 1 |
| IL19-3234 | Pr_Urb_21 | 1 |
| IL19-3242 | Pr_Urb_21 | 1 |
| IL19-325 | Pr_Urb_21, Adv_Urb_22 | 2 |
| IL19-3278 | Pr_Urb_21, AE_Urb_22 | 2 |
| IL19-3328 | Pr_Urb_21 | 1 |
| IL19-3464 | Pr_Urb_21 | 1 |
| IL19-3613 | Pr_Urb_21, Adv_Urb_22 | 2 |
| IL19-3618 | Pr_Urb_21, Adv_Urb_22 | 2 |
| IL19-3627 | Pr_Urb_21 | 1 |
| IL19-3632 | Pr_Urb_21, Adv_Urb_22 | 2 |
| IL19-3661 | Pr_Urb_21, AE_Urb_22 | 2 |
| IL19-3723 | Pr_Urb_21 | 1 |
| IL19-3765 | Pr_Urb_21 | 1 |
| IL19-3852 | Pr_Urb_21 | 1 |
| IL19-3857 | Pr_Urb_21 | 1 |
| IL19-3862 | Pr_Urb_21 | 1 |
| IL19-3877 | Pr_Urb_21, AE_Urb_22 | 2 |
| IL19-3935 | Pr_Urb_21 | 1 |
| IL19-3937 | Pr_Urb_21 | 1 |
| IL19-3975 | Pr_Urb_21 | 1 |
| IL19-3978 | Pr_Urb_21, Adv_Urb_22 | 2 |
| IL19-4009 | Pr_Urb_21 | 1 |
| IL19-4010 | Pr_Urb_21 | 1 |
| IL19-4046 | Pr_Urb_21, AE_Urb_22 | 2 |
| IL19-4048 | Pr_Urb_21 | 1 |
| IL19-4062 | Pr_Urb_21, Adv_Urb_22 | 2 |
| IL19-4066 | Pr_Urb_21 | 1 |
| IL19-4070 | Pr_Urb_21 | 1 |
| IL19-4076 | Pr_Urb_21 | 1 |
| IL19-409 | Pr_Urb_21, Adv_Urb_22 | 2 |
| IL19-410 | Pr_Urb_21 | 1 |
| IL19-4124 | Pr_Urb_21, AE_Urb_22 | 2 |
| IL19-4163 | Pr_Urb_21 | 1 |
| IL19-4174 | Pr_Urb_21 | 1 |
| IL19-418 | Pr_Urb_21 | 1 |
| IL19-4279 | Pr_Urb_21 | 1 |
| IL19-4305 | Pr_Urb_21 | 1 |
| IL19-4312 | Pr_Urb_21 | 1 |
| IL19-4427 | Pr_Urb_21 | 1 |
| IL19-4429 | Pr_Urb_21, Adv_Urb_22 | 2 |
| IL19-4442 | Pr_Urb_21 | 1 |
| IL19-4539 | Pr_Urb_21 | 1 |
| IL19-4544 | Pr_Urb_21, AE_Urb_22 | 2 |
| IL19-4551 | Pr_Urb_21 | 1 |
| IL19-4556 | Pr_Urb_21, Adv_Urb_22 | 2 |
| IL19-4563 | Pr_Urb_21, AE_Urb_22 | 2 |
| IL19-4565 | Pr_Urb_21 | 1 |
| IL19-4567 | Pr_Urb_21 | 1 |
| IL19-4573 | Pr_Urb_21 | 1 |
| IL19-4632 | Pr_Urb_21 | 1 |
| IL19-4672 | Pr_Urb_21 | 1 |
| IL19-4679 | Pr_Urb_21 | 1 |
| IL19-4720 | Pr_Urb_21 | 1 |
| IL19-473 | Pr_Urb_21 | 1 |
| IL19-4737 | Pr_Urb_21 | 1 |
| IL19-474 | Pr_Urb_21 | 1 |
| IL19-4743 | Pr_Urb_21 | 1 |
| IL19-4747 | Pr_Urb_21 | 1 |
| IL19-4771 | Pr_Urb_21 | 1 |
| IL19-4804 | Pr_Urb_21 | 1 |
| IL19-4811 | Pr_Urb_21 | 1 |
| IL19-4820 | Pr_Urb_21, Adv_Urb_22 | 2 |
| IL19-4833 | Pr_Urb_21, Adv_Urb_22 | 2 |
| IL19-4848 | Pr_Urb_21 | 1 |
| IL19-4853 | Pr_Urb_21, Adv_Urb_22 | 2 |
| IL19-4873 | Pr_Urb_21 | 1 |
| IL19-4877 | Pr_Urb_21, Adv_Urb_22 | 2 |
| IL19-5010 | Pr_Urb_21 | 1 |
| IL19-5056 | Pr_Urb_21 | 1 |
| IL19-5074 | Pr_Urb_21, Adv_Urb_22 | 2 |
| IL19-5107 | Pr_Urb_21 | 1 |
| IL19-511 | Pr_Urb_21, AE_Urb_22 | 2 |
| IL19-5161 | Pr_Urb_21 | 1 |
| IL19-522 | Pr_Urb_21 | 1 |
| IL19-5220 | Pr_Urb_21 | 1 |
| IL19-5242 | Pr_Urb_21, Adv_Urb_22 | 2 |
| IL19-5278 | Pr_Urb_21 | 1 |
| IL19-5332 | Pr_Urb_21, Adv_Urb_22 | 2 |
| IL19-5336 | Pr_Urb_21, Adv_Urb_22 | 2 |
| IL19-5362 | Pr_Urb_21, AE_Urb_22 | 2 |
| IL19-5437 | Pr_Urb_21 | 1 |
| IL19-5455 | Pr_Urb_21 | 1 |
| IL19-5467 | Pr_Urb_21, Adv_Urb_22 | 2 |
| IL19-5502 | Pr_Urb_21, Adv_Urb_22 | 2 |
| IL19-5553 | Pr_Urb_21 | 1 |
| IL19-5564 | Pr_Urb_21 | 1 |
| IL19-5603 | Pr_Urb_21 | 1 |
| IL19-5610 | Pr_Urb_21 | 1 |
| IL19-5626 | Pr_Urb_21 | 1 |
| IL19-5628 | Pr_Urb_21 | 1 |
| IL19-5630 | Pr_Urb_21 | 1 |
| IL19-5644 | Pr_Urb_21 | 1 |
| IL19-5645 | Pr_Urb_21 | 1 |
| IL19-5657 | Pr_Urb_21 | 1 |
| IL19-5679 | Pr_Urb_21 | 1 |
| IL19-5703 | Pr_Urb_21, Adv_Urb_22 | 2 |
| IL19-5704 | Pr_Urb_21, Adv_Urb_22 | 2 |
| IL19-5718 | Pr_Urb_21, Adv_Urb_22 | 2 |
| IL19-5723 | Pr_Urb_21, Adv_Urb_22 | 2 |
| IL19-5737 | Pr_Urb_21, Adv_Urb_22 | 2 |
| IL19-5761 | Pr_Urb_21 | 1 |
| IL19-5801 | Pr_Urb_21 | 1 |
| IL19-5820 | Pr_Urb_21 | 1 |
| IL19-6017 | Pr_Urb_21 | 1 |
| IL19-6019 | Pr_Urb_21, Adv_Urb_22 | 2 |
| IL19-6023 | Pr_Urb_21, Adv_Urb_22 | 2 |
| IL19-6028 | Pr_Urb_21 | 1 |
| IL19-6031 | Pr_Urb_21, Adv_Urb_22 | 2 |
| IL19-6035 | Pr_Urb_21, Adv_Urb_22 | 2 |
| IL19-6064 | Pr_Urb_21 | 1 |
| IL19-6066 | Pr_Urb_21 | 1 |
| IL19-6121 | Pr_Urb_21 | 1 |
| IL19-6128 | Pr_Urb_21 | 1 |
| IL19-6130 | Pr_Urb_21 | 1 |
| IL19-6164 | Pr_Urb_21, AE_Urb_22 | 2 |
| IL19-6168 | Pr_Urb_21 | 1 |
| IL19-6203 | Pr_Urb_21 | 1 |
| IL19-6204 | Pr_Urb_21 | 1 |
| IL19-6259 | Pr_Urb_21 | 1 |
| IL19-6277 | Pr_Urb_21 | 1 |
| IL19-6278 | Pr_Urb_21, Adv_Urb_22 | 2 |
| IL19-6345 | Pr_Urb_21, Adv_Urb_22 | 2 |
| IL19-6406 | Pr_Urb_21, Adv_Urb_22 | 2 |
| IL19-642 | Pr_Urb_21 | 1 |
| IL19-6424 | Pr_Urb_21 | 1 |
| IL19-643 | Pr_Urb_21 | 1 |
| IL19-6432 | Pr_Urb_21, Adv_Urb_22 | 2 |
| IL19-6441 | Pr_Urb_21 | 1 |
| IL19-6446 | Pr_Urb_21 | 1 |
| IL19-6449 | Pr_Urb_21 | 1 |
| IL19-6468 | Pr_Urb_21, AE_Urb_22 | 2 |
| IL19-6529 | Pr_Urb_21, Adv_Urb_22 | 2 |
| IL19-6537 | Pr_Urb_21, AE_Urb_22 | 2 |
| IL19-6556 | Pr_Urb_21 | 1 |
| IL19-6561 | Pr_Urb_21, AE_Urb_22 | 2 |
| IL19-6568 | Pr_Urb_21 | 1 |
| IL19-6571 | Pr_Urb_21 | 1 |
| IL19-6575 | Pr_Urb_21 | 1 |
| IL19-6605 | Pr_Urb_21, Adv_Urb_22 | 2 |
| IL19-6633 | Pr_Urb_21 | 1 |
| IL19-6634 | Pr_Urb_21 | 1 |
| IL19-6636 | Pr_Urb_21 | 1 |
| IL19-6637 | Pr_Urb_21 | 1 |
| IL19-6642 | Pr_Urb_21 | 1 |
| IL19-6644 | Pr_Urb_21, AE_Urb_22 | 2 |
| IL19-6667 | Pr_Urb_21, Adv_Urb_22 | 2 |
| IL19-6715 | Pr_Urb_21 | 1 |
| IL19-6720 | Pr_Urb_21 | 1 |
| IL19-677 | Pr_Urb_21, Adv_Urb_22 | 2 |
| IL19-6860 | Pr_Urb_21, Adv_Urb_22 | 2 |
| IL19-6867 | Pr_Urb_21 | 1 |
| IL19-6868 | Pr_Urb_21, Adv_Urb_22 | 2 |
| IL19-6871 | Pr_Urb_21 | 1 |
| IL19-6913 | Pr_Urb_21, Adv_Urb_22 | 2 |
| IL19-6920 | Pr_Urb_21, AE_Urb_22 | 2 |
| IL19-6955 | Pr_Urb_21, AE_Urb_22 | 2 |
| IL19-6972 | Pr_Urb_21, AE_Urb_22 | 2 |
| IL19-6979 | Pr_Urb_21, Adv_Urb_22 | 2 |
| IL19-7002 | Pr_Urb_21, AE_Urb_22 | 2 |
| IL19-7003 | Pr_Urb_21 | 1 |
| IL19-7004 | Pr_Urb_21 | 1 |
| IL19-7007 | Pr_Urb_21, AE_Urb_22 | 2 |
| IL19-7008 | Pr_Urb_21, AE_Urb_22 | 2 |
| IL19-7016 | Pr_Urb_21 | 1 |
| IL19-7020 | Pr_Urb_21 | 1 |
| IL19-7048 | Pr_Urb_21, AE_Urb_22 | 2 |
| IL19-7058 | Pr_Urb_21, AE_Urb_22 | 2 |
| IL19-7061 | Pr_Urb_21 | 1 |
| IL19-7063 | Pr_Urb_21, AE_Urb_22 | 2 |
| IL19-7076 | Pr_Urb_21 | 1 |
| IL19-7101 | Pr_Urb_21, Adv_Urb_22 | 2 |
| IL19-7105 | Pr_Urb_21 | 1 |
| IL19-7112 | Pr_Urb_21 | 1 |
| IL19-7230 | Pr_Urb_21 | 1 |
| IL19-7247 | Pr_Urb_21 | 1 |
| IL19-7248 | Pr_Urb_21 | 1 |
| IL19-725 | Pr_Urb_21 | 1 |
| IL19-7256 | Pr_Urb_21 | 1 |
| IL19-7260 | Pr_Urb_21 | 1 |
| IL19-7271 | Pr_Urb_21 | 1 |
| IL19-7311 | Pr_Urb_21, Adv_Urb_22 | 2 |
| IL19-744 | Pr_Urb_21 | 1 |
| IL19-7518 | Pr_Urb_21 | 1 |
| IL19-753 | Pr_Urb_21 | 1 |
| IL19-803 | Pr_Urb_21 | 1 |
| IL19-957 | Pr_Urb_21 | 1 |
| IL19-964 | Pr_Urb_21 | 1 |
| IL19-971 | Pr_Urb_21, AE_Urb_22 | 2 |
| IL20-1031 | Pr_Urb_22 | 1 |
| IL20-1037 | Pr_Urb_22 | 1 |
| IL20-1045 | Pr_Urb_22 | 1 |
| IL20-1138 | Pr_Urb_22 | 1 |
| IL20-1234 | Pr_Urb_22 | 1 |
| IL20-1243 | Pr_Urb_22 | 1 |
| IL20-1249 | Pr_Urb_22 | 1 |
| IL20-1258 | Pr_Urb_22 | 1 |
| IL20-127 | Pr_Urb_22 | 1 |
| IL20-1365 | Pr_Urb_22 | 1 |
| IL20-1415 | Pr_Urb_22 | 1 |
| IL20-144 | Pr_Urb_22 | 1 |
| IL20-1461 | Pr_Urb_22 | 1 |
| IL20-1465 | Pr_Urb_22 | 1 |
| IL20-1513 | Pr_Urb_22 | 1 |
| IL20-1527 | Pr_Urb_22 | 1 |
| IL20-1536 | Pr_Urb_22 | 1 |
| IL20-1613 | Pr_Urb_22 | 1 |
| IL20-1629 | Pr_Urb_22 | 1 |
| IL20-1751 | Pr_Urb_22 | 1 |
| IL20-177 | Pr_Urb_22 | 1 |
| IL20-195 | Pr_Urb_22 | 1 |
| IL20-1960 | Pr_Urb_22 | 1 |
| IL20-1976 | Pr_Urb_22 | 1 |
| IL20-2134 | Pr_Urb_22 | 1 |
| IL20-2142 | Pr_Urb_22 | 1 |
| IL20-216 | Pr_Urb_22 | 1 |
| IL20-2169 | Pr_Urb_22 | 1 |
| IL20-2218 | Pr_Urb_22 | 1 |
| IL20-2235 | Pr_Urb_22 | 1 |
| IL20-2418 | Pr_Urb_22 | 1 |
| IL20-242 | Pr_Urb_22 | 1 |
| IL20-2466 | Pr_Urb_22 | 1 |
| IL20-2675 | Pr_Urb_22 | 1 |
| IL20-2737 | Pr_Urb_22 | 1 |
| IL20-2762 | Pr_Urb_22 | 1 |
| IL20-2767 | Pr_Urb_22 | 1 |
| IL20-2944 | Pr_Urb_22 | 1 |
| IL20-2946 | Pr_Urb_22 | 1 |
| IL20-2950 | Pr_Urb_22 | 1 |
| IL20-2972 | Pr_Urb_22 | 1 |
| IL20-3017 | Pr_Urb_22 | 1 |
| IL20-3031 | Pr_Urb_22 | 1 |
| IL20-3067 | Pr_Urb_22 | 1 |
| IL20-3111 | Pr_Urb_22 | 1 |
| IL20-3119 | Pr_Urb_22 | 1 |
| IL20-3120 | Pr_Urb_22 | 1 |
| IL20-3142 | Pr_Urb_22 | 1 |
| IL20-3177 | Pr_Urb_22 | 1 |
| IL20-3273 | Pr_Urb_22 | 1 |
| IL20-3318 | Pr_Urb_22 | 1 |
| IL20-335 | Pr_Urb_22 | 1 |
| IL20-339 | Pr_Urb_22 | 1 |
| IL20-3425 | Pr_Urb_22 | 1 |
| IL20-3443 | Pr_Urb_22 | 1 |
| IL20-3534 | Pr_Urb_22 | 1 |
| IL20-3565 | Pr_Urb_22 | 1 |
| IL20-3574 | Pr_Urb_22 | 1 |
| IL20-3619 | Pr_Urb_22 | 1 |
| IL20-3658 | Pr_Urb_22 | 1 |
| IL20-371 | Pr_Urb_22 | 1 |
| IL20-3715 | Pr_Urb_22 | 1 |
| IL20-3748 | Pr_Urb_22 | 1 |
| IL20-3749 | Pr_Urb_22 | 1 |
| IL20-3869 | Pr_Urb_22 | 1 |
| IL20-3929 | Pr_Urb_22 | 1 |
| IL20-395 | Pr_Urb_22 | 1 |
| IL20-3969 | Pr_Urb_22 | 1 |
| IL20-4027 | Pr_Urb_22 | 1 |
| IL20-4031 | Pr_Urb_22 | 1 |
| IL20-4032 | Pr_Urb_22 | 1 |
| IL20-4036 | Pr_Urb_22 | 1 |
| IL20-4153 | Pr_Urb_22 | 1 |
| IL20-4156 | Pr_Urb_22 | 1 |
| IL20-4157 | Pr_Urb_22 | 1 |
| IL20-4162 | Pr_Urb_22 | 1 |
| IL20-4164 | Pr_Urb_22 | 1 |
| IL20-4210 | Pr_Urb_22 | 1 |
| IL20-422 | Pr_Urb_22 | 1 |
| IL20-4231 | Pr_Urb_22 | 1 |
| IL20-424 | Pr_Urb_22 | 1 |
| IL20-4241 | Pr_Urb_22 | 1 |
| IL20-426 | Pr_Urb_22 | 1 |
| IL20-4268 | Pr_Urb_22 | 1 |
| IL20-4332 | Pr_Urb_22 | 1 |
| IL20-4356 | Pr_Urb_22 | 1 |
| IL20-4361 | Pr_Urb_22 | 1 |
| IL20-4371 | Pr_Urb_22 | 1 |
| IL20-438 | Pr_Urb_22 | 1 |
| IL20-442 | Pr_Urb_22 | 1 |
| IL20-4455 | Pr_Urb_22 | 1 |
| IL20-4457 | Pr_Urb_22 | 1 |
| IL20-4461 | Pr_Urb_22 | 1 |
| IL20-4477 | Pr_Urb_22 | 1 |
| IL20-4528 | Pr_Urb_22 | 1 |
| IL20-456 | Pr_Urb_22 | 1 |
| IL20-463 | Pr_Urb_22 | 1 |
| IL20-4642 | Pr_Urb_22 | 1 |
| IL20-467 | Pr_Urb_22 | 1 |
| IL20-4720 | Pr_Urb_22 | 1 |
| IL20-4732 | Pr_Urb_22 | 1 |
| IL20-4746 | Pr_Urb_22 | 1 |
| IL20-475 | Pr_Urb_22 | 1 |
| IL20-4752 | Pr_Urb_22 | 1 |
| IL20-4812 | Pr_Urb_22 | 1 |
| IL20-5141 | Pr_Urb_22 | 1 |
| IL20-5144 | Pr_Urb_22 | 1 |
| IL20-5152 | Pr_Urb_22 | 1 |
| IL20-522 | Pr_Urb_22 | 1 |
| IL20-5269 | Pr_Urb_22 | 1 |
| IL20-5316 | Pr_Urb_22 | 1 |
| IL20-534 | Pr_Urb_22 | 1 |
| IL20-5351 | Pr_Urb_22 | 1 |
| IL20-5353 | Pr_Urb_22 | 1 |
| IL20-544 | Pr_Urb_22 | 1 |
| IL20-5465 | Pr_Urb_22 | 1 |
| IL20-5565 | Pr_Urb_22 | 1 |
| IL20-5577 | Pr_Urb_22 | 1 |
| IL20-5618 | Pr_Urb_22 | 1 |
| IL20-5633 | Pr_Urb_22 | 1 |
| IL20-5717 | Pr_Urb_22 | 1 |
| IL20-5913 | Pr_Urb_22 | 1 |
| IL20-597 | Pr_Urb_22 | 1 |
| IL20-6029 | Pr_Urb_22 | 1 |
| IL20-6069 | Pr_Urb_22 | 1 |
| IL20-6115 | Pr_Urb_22 | 1 |
| IL20-615 | Pr_Urb_22 | 1 |
| IL20-618 | Pr_Urb_22 | 1 |
| IL20-6262 | Pr_Urb_22 | 1 |
| IL20-6346 | Pr_Urb_22 | 1 |
| IL20-6350 | Pr_Urb_22 | 1 |
| IL20-646 | Pr_Urb_22 | 1 |
| IL20-6468 | Pr_Urb_22 | 1 |
| IL20-6545 | Pr_Urb_22 | 1 |
| IL20-6671 | Pr_Urb_22 | 1 |
| IL20-6737 | Pr_Urb_22 | 1 |
| IL20-6746 | Pr_Urb_22 | 1 |
| IL20-6749 | Pr_Urb_22 | 1 |
| IL20-677 | Pr_Urb_22 | 1 |
| IL20-678 | Pr_Urb_22 | 1 |
| IL20-6928 | Pr_Urb_22 | 1 |
| IL20-7013 | Pr_Urb_22 | 1 |
| IL20-7020 | Pr_Urb_22 | 1 |
| IL20-7025 | Pr_Urb_22 | 1 |
| IL20-7029 | Pr_Urb_22 | 1 |
| IL20-7033 | Pr_Urb_22 | 1 |
| IL20-7050 | Pr_Urb_22 | 1 |
| IL20-7067 | Pr_Urb_22 | 1 |
| IL20-711 | Pr_Urb_22 | 1 |
| IL20-7123 | Pr_Urb_22 | 1 |
| IL20-7124 | Pr_Urb_22 | 1 |
| IL20-7126 | Pr_Urb_22 | 1 |
| IL20-7157 | Pr_Urb_22 | 1 |
| IL20-7233 | Pr_Urb_22 | 1 |
| IL20-7266 | Pr_Urb_22 | 1 |
| IL20-729 | Pr_Urb_22 | 1 |
| IL20-7420 | Pr_Urb_22 | 1 |
| IL20-745 | Pr_Urb_22 | 1 |
| IL20-746 | Pr_Urb_22 | 1 |
| IL20-748 | Pr_Urb_22 | 1 |
| IL20-7544 | Pr_Urb_22 | 1 |
| IL20-761 | Pr_Urb_22 | 1 |
| IL20-7655 | Pr_Urb_22 | 1 |
| IL20-7725 | Pr_Urb_22 | 1 |
| IL20-7727 | Pr_Urb_22 | 1 |
| IL20-7735 | Pr_Urb_22 | 1 |
| IL20-7746 | Pr_Urb_22 | 1 |
| IL20-7823 | Pr_Urb_22 | 1 |
| IL20-7841 | Pr_Urb_22 | 1 |
| IL20-7852 | Pr_Urb_22 | 1 |
| IL20-7862 | Pr_Urb_22 | 1 |
| IL20-7863 | Pr_Urb_22 | 1 |
| IL20-7914 | Pr_Urb_22 | 1 |
| IL20-797 | Pr_Urb_22 | 1 |
| IL20-815 | Pr_Urb_22 | 1 |
| IL20-8178 | Pr_Urb_22 | 1 |
| IL20-8179 | Pr_Urb_22 | 1 |
| IL20-8211 | Pr_Urb_22 | 1 |
| IL20-8214 | Pr_Urb_22 | 1 |
| IL20-8217 | Pr_Urb_22 | 1 |
| IL20-8221 | Pr_Urb_22 | 1 |
| IL20-823 | Pr_Urb_22 | 1 |
| IL20-8233 | Pr_Urb_22 | 1 |
| IL20-8234 | Pr_Urb_22 | 1 |
| IL20-8244 | Pr_Urb_22 | 1 |
| IL20-825 | Pr_Urb_22 | 1 |
| IL20-8277 | Pr_Urb_22 | 1 |
| IL20-8320 | Pr_Urb_22 | 1 |
| IL20-8342 | Pr_Urb_22 | 1 |
| IL20-8345 | Pr_Urb_22 | 1 |
| IL20-8368 | Pr_Urb_22 | 1 |
| IL20-8372 | Pr_Urb_22 | 1 |
| IL20-8434 | Pr_Urb_22 | 1 |
| IL20-8446 | Pr_Urb_22 | 1 |
| IL20-8456 | Pr_Urb_22 | 1 |
| IL20-8462 | Pr_Urb_22 | 1 |
| IL20-8515 | Pr_Urb_22 | 1 |
| IL20-856 | Pr_Urb_22 | 1 |
| IL20-98 | Pr_Urb_22 | 1 |
| NATTY | Drl_Urb_21, Drl_Urb_22 | 2 |
| OGLE | Drl_Urb_21, Drl_Urb_22 | 2 |
| P02A17815 | Drl_Urb_22 | 1 |
| REINS | Adv_Urb_21, AE_Urb_21, Drl_Urb_21, Pr_Urb_21, Adv_Urb_22, AE_Urb_22, Drl_Urb_22 | 7 |
| RUSHMORE | Drl_Urb_21, Drl_Urb_22 | 2 |
| SABER | Adv_Urb_21, AE_Urb_21, Drl_Urb_21, Pr_Urb_21, Adv_Urb_22, AE_Urb_22, Drl_Urb_22 | 7 |
| SADDLE | Drl_Urb_21, Drl_Urb_22 | 2 |
| SHELBY427 | Drl_Urb_22 | 1 |
| SPURS | Adv_Urb_21, AE_Urb_21, Drl_Urb_21, Pr_Urb_21, Adv_Urb_22, AE_Urb_22, Drl_Urb_22 | 7 |

*Accessions are in alphabetic order.

Supplementary Table 2: Descriptive summary for the BLUPs of different traits (primary milling and secondary seed morphometric Traits) in years 2021, 2022, and combined.

|  | 2021 | | | 2022 | | | Combined | | |
| --- | --- | --- | --- | --- | --- | --- | --- | --- | --- |
| Trait | Mean | Min | Max | Mean | Min | Max | Mean | Min | Max |
| Test Weight (g/L) | 404.0 | 337.9 | 454.6 | 344.8 | 272.8 | 412.9 | 377.3 | 333.1 | 425.5 |
| Thins Percentage (%) | 14.4 | 2.5 | 49.5 | 10.5 | 2.8 | 30.2 | 13.1 | 4.7 | 36.9 |
| Groat Percentage (%) | 69.2 | 60.2 | 77.2 | 65.3 | 55.5 | 70.7 | 67.5 | 61.4 | 72.3 |
| Kernel Length (mm) | 10.6 | 8.4 | 12.0 | 10.9 | 9.5 | 12.2 | 10.7 | 8.6 | 12.2 |
| Kernel Width (mm) | 2.5 | 2.3 | 2.8 | 2.5 | 2.4 | 2.8 | 2.5 | 2.3 | 2.8 |
| Kernel Perimeter (mm) | 26.3 | 21.9 | 29.9 | 27.2 | 23.5 | 29.8 | 26.7 | 22.5 | 30.3 |
| Kernel Area (mm^2^) | 20.9 | 16.5 | 24.2 | 21.4 | 17.9 | 24.5 | 21.1 | 16.9 | 24.2 |
| Groat Length (mm) | 6.4 | 5.7 | 7.1 | 7.2 | 6.3 | 7.9 | 6.7 | 5.9 | 7.4 |
| Groat Width (mm) | 2.4 | 2.1 | 2.6 | 2.4 | 2.3 | 2.6 | 2.4 | 2.2 | 2.6 |
| Groat Perimeter (mm) | 15.0 | 13.8 | 16.3 | 16.7 | 15.8 | 17.6 | 15.7 | 14.4 | 16.9 |
| Groat Area (mm^2^) | 11.9 | 10.3 | 13.6 | 13.5 | 12.0 | 14.8 | 12.6 | 11.1 | 14.0 |

Supplementary Table 3: Wald test result of primary and secondary traits across the years.

|  | Test weight | | | Thins Percentage | | | Groat Percentage | | |  |  |  |
| --- | --- | --- | --- | --- | --- | --- | --- | --- | --- | --- | --- | --- |
|  | Sums of Square | Wald Stat. | Pr (Chisq) | Sums of Square | Wald Stat. | Pr (Chisq) | Sums of Square | Wald Stat. | Pr (Chisq) |  |  |  |
| Germplasm | 1071095.29 | 7117.95 | <0.001 | 31549.96 | 5945.44 | <0.001 | 8754.11 | 2983.98 | <0.001 |  |  |  |
| Year | 29440.34 | 195.65 | <0.001 | 8.19 | 1.54 | 0.21 | 7.59 | 2.59 | 0.11 |  |  |  |
| Germplasm: Year | 155609.99 | 1034.1 | <0.001 | 3773.63 | 711.12 | <0.001 | 1921 | 654.81 | <0.001 |  |  |  |
|  |  |  |  |  |  |  |  |  |  |  |  |  |
|  | Kernel Length | | | Kernel Width | | | Kernel Perimeter | | | Kernel Area | | |
|  | Sums of Square | Wald Stat. | Pr (Chisq) | Sums of Square | Wald Stat. | Pr (Chisq) | Sums of Square | Wald  Stat. | Pr (Chisq) | Sums of Square | Wald  Stat. | Pr (Chisq) |
| Germplasm | 559.57 | 3159.07 | <0.001 | 22.1 | 2803.43 | <0.001 | 3773.71 | 2637.76 | <0.001 | 4405.03 | 2450.47 | <0.001 |
| Year | 0.51 | 2.88 | 0.09 | 0 | 0.13 | 0.72 | 2.22 | 1.55 | 0.21 | 0.3 | 0.16 | 0.68 |
| Germplasm: Year | 33.1 | 186.86 | 0.01 | 1.47 | 186.89 | 0.01 | 254.82 | 178.12 | 0.04 | 350.39 | 194.92 | 0 |
|  |  |  |  |  |  |  |  |  |  |  |  |  |
|  | Groat Length | | | Groat Width | | | Groat Perimeter | | | Groat Area | | |
|  | Sums of Square | Wald Stat. | Pr (Chisq) | Sums of Square | Wald Stat. | Pr (Chisq) | Sums of Square | Wald  Stat. | Pr (Chisq) | Sums of Square | Wald  Stat. | Pr (Chisq) |
| Germplasm | 157.48 | 3067.1 | <0.001 | 14.97 | 3382.08 | <0.001 | 605.11 | 2494.74 | <0.001 | 928.46 | 2331.14 | <0.001 |
| Year | 4.56 | 88.88 | <0.001 | 0.01 | 1.3 | 0.26 | 5.31 | 21.88 | <0.001 | 12.31 | 30.91 | <0.001 |
| Germplasm: Year | 11.11 | 216.45 | <0.001 | 0.97 | 218.79 | <0.001 | 62.27 | 256.73 | <0.001 | 87.49 | 219.67 | <0.001 |

Supplementary Table 4: Descriptive statistics of different cross-validation schemes and their comparison to ST-CV1 for primary milling traits in 2021, 2022, and across both years.

|  |  | **2021 Season** | | | | **2022 Season** | | | | **Across seasons** | | | | |
| --- | --- | --- | --- | --- | --- | --- | --- | --- | --- | --- | --- | --- | --- | --- |
| **Trait** | **Model** | **Prediction accuracy** | | | **Superiority  over ST-CV1** | **Prediction accuracy** | | | **Superiority  over ST-CV1** | **Prediction accuracy** | | | **Superiority  over ST-CV1** |  |
|  |  | **Mean** | **Min.** | **Max.** |  | **Mean** | **Min.** | **Max.** |  | **Mean** | **Min.** | **Max.** |  |  |
| **Test Weight** | **ST-CV1** | 0.32 | 0.01 | 0.49 | - | 0.49 | 0.35 | 0.63 | - | 0.39 | 0.23 | 0.49 | - |  |
|  | **MT-CV1** | 0.44 | 0.24 | 0.66 | 37.4 | 0.53 | 0.37 | 0.72 | 6.69 | 0.48 | 0.36 | 0.59 | 24.19 |  |
|  | **MT-CV2** | 0.54 | 0.37 | 0.71 | 67.82 | 0.64 | 0.51 | 0.77 | 28.91 | 0.59 | 0.49 | 0.7 | 52.1 |  |
|  | **MT-CV3** | 0.44 | 0.24 | 0.67 | 37.4 | 0.53 | 0.36 | 0.7 | 7.79 | 0.48 | 0.37 | 0.58 | 24.81 |  |
|  | **MT-CV4** | 0.45 | 0.25 | 0.68 | 40.85 | 0.57 | 0.42 | 0.69 | 14.92 | 0.52 | 0.39 | 0.62 | 33.62 |  |
|  | **MT-CV5** | 0.41 | 0.24 | 0.58 | 27.95 | 0.53 | 0.43 | 0.69 | 6.68 | 0.19 | 0.05 | 0.33 | -50.76 |  |
|  | **MT-CV6** | 0.43 | 0.24 | 0.67 | 34.72 | 0.63 | 0.47 | 0.75 | 28.16 | 0.53 | 0.4 | 0.67 | 36.18 |  |
| **Thins Percentage** | **ST-CV1** | 0.38 | 0.22 | 0.46 | - | 0.38 | 0.22 | 0.55 | - | 0.38 | 0.23 | 0.46 | - |  |
|  | **MT-CV1** | 0.46 | 0.39 | 0.57 | 19.97 | 0.43 | 0.23 | 0.61 | 14.04 | 0.46 | 0.28 | 0.59 | 22.04 |  |
|  | **MT-CV2** | 0.61 | 0.52 | 0.7 | 59.2 | 0.6 | 0.44 | 0.71 | 59.28 | 0.6 | 0.47 | 0.71 | 59.16 |  |
|  | **MT-CV3** | 0.44 | 0.31 | 0.55 | 14.48 | 0.41 | 0.19 | 0.58 | 10.32 | 0.46 | 0.27 | 0.62 | 20.68 |  |
|  | **MT-CV4** | 0.61 | 0.52 | 0.68 | 57.4 | 0.48 | 0.14 | 0.61 | 27.97 | 0.55 | 0.39 | 0.68 | 44.97 |  |
|  | **MT-CV5** | 0.47 | 0.27 | 0.62 | 21.49 | 0.52 | 0.39 | 0.65 | 39.04 | 0.49 | 0.39 | 0.69 | 30.71 |  |
|  | **MT-CV6** | 0.57 | 0.46 | 0.65 | 47.41 | 0.53 | 0.37 | 0.69 | 41.72 | 0.59 | 0.46 | 0.72 | 55.45 |  |
| **Groat Percentage** | **ST-CV1** | 0.21 | 0.07 | 0.39 | - | 0.49 | 0.29 | 0.65 | - | 0.4 | 0.28 | 0.54 | - |  |
|  | **MT-CV1** | 0.27 | 0.13 | 0.46 | 27.03 | 0.47 | 0.28 | 0.63 | -3.93 | 0.44 | 0.27 | 0.58 | 9.17 |  |
|  | **MT-CV2** | 0.27 | 0.13 | 0.45 | 25.31 | 0.48 | 0.32 | 0.64 | -2.31 | 0.43 | 0.28 | 0.57 | 6.21 |  |
|  | **MT-CV3** | 0.29 | 0.14 | 0.47 | 33.13 | 0.46 | 0.26 | 0.65 | -5.73 | 0.44 | 0.26 | 0.57 | 10.51 |  |
|  | **MT-CV4** | 0.3 | 0.07 | 0.46 | 39.92 | 0.47 | 0.31 | 0.61 | -4.3 | 0.45 | 0.26 | 0.58 | 11.92 |  |
|  | **MT-CV5** | 0.02 | -0.21 | 0.2 | -91.9 | -0.03 | -0.34 | 0.2 | -106.86 | -0.08 | -0.17 | 0.08 | -119.13 |  |
|  | **MT-CV6** | 0.13 | -0.21 | 0.34 | -39.25 | 0.38 | 0.28 | 0.6 | -21.88 | 0.35 | 0.18 | 0.56 | -13.28 |  |
