# Supplementary material for: Implementing multi‐trait genomic selection to improve grain milling quality in oats (Avena sativa L.)
Source: Plant Genome. 2024 May 19;17(2):e20457. doi: 10.1002/tpg2.20457 (PMC12807195; doi:10.1002/tpg2.20457)
Supplement: Supplementary file 2 [file TPG2-17-e20457-s002.docx]

**SUPPLEMENTARY FIGURES**

**Implementing multi-trait genomic selection to improve grain milling quality in oats (*Avena sativa* L.)**

**Anup Dhakal, Jesse Poland, Laxman Adhikari, Ethan Faryna, Jason Fiedler, Jessica E. Rutkoski, and Juan David Arbelaez.**

**Supplementary Figure 1:** UIUC Oat breeding Pipeline. The early stage of yield trial is preliminary yield trial where ~300 lines are advanced from the augumented trial. This trial is followed by advanced and advanced early trial and drill trials. Advanced and Drill trials are multi-location trials. In existing pipelines, the lines are phenotyped for milling traits and selected as parents only in year 5. However, with MTGS, the parents can be selected at year three or year four, reducing the breeding cycle.

**Supplementary Figure 2:** Research workflow, chronological order, and how the primary and secondary traits were measured.

**Supplementary Figure 3:** Distribution of the primary milling and secondary morphometric traits in the year 2021.

**Supplementary Figure 4:** Distribution of the primary milling and secondary morphometric traits in the year 2022.

**Supplementary Figure 5**: Distribution of the primary milling and secondary morphometric traits across 2021 and 2022 combined.

**Supplementary Figure 6:** Population structure among all the genotypes tested in the study.

**Supplementary Figure 7:** Broad sense heritability (H^2^) in the vertical column and genetic correlation between the primary grain milling quality traits and secondary grain morphometric traits in year 2021.

**Supplementary Figure 8:** Broad sense heritability (H^2^) in the vertical column and genetic correlation between the primary grain milling quality traits and secondary grain morphometric traits in year 2022.

**Supplementary Figure 9:** Manhattan plot and QQplot of genome-wide association study (GWAS) using 2148 SNPs for three milling traits evaluated between 2021 and 2022.

**Supplementary Figure 9 (continue):** Manhattan plot and QQplot of genome-wide association study (GWAS) using 2148 SNPs for four kernel morphometric milling traits evaluated between 2021 and 2022.

**Supplementary Figure 9 (continue):** Manhattan plot and QQplot of genome-wide association study (GWAS) using 2148 SNPs for four groat morphometric milling traits evaluated between 2021 and 2022.

**Supplementary Figure 10:** Prediction accuracy of seven cross-validation models for the three milling traits (Test weight, Thins Percentage, and Groat Percentage) in 2021. STCV1; Single-trait Genomic Prediction, MT-CV1; Multi-trait Genomic Prediction (MT) without secondary trait from the kernels in the candidate set, MT-CV2; MT with secondary traits from kernels (MT-CV2), MT-CV3; Multi-trait Genomic Prediction (MT) without secondary trait from the groat in the candidate set, MT-CV4: Multi-trait Genomic Prediction (MT) with secondary trait from the groat in the candidate set, MT-CV5; MT with secondary traits from kernels but without marker information, MT-CV6: Multi-trait Genomic Prediction (MT) with secondary trait from the kernels in the candidate set but phenotypic information available is unreplicated (replication 1 only).

**Supplementary Figure 11:** Prediction accuracy of seven different cross-validation models for the three milling traits (Test weight, Thins Percentage, and Groat Percentage) in the year 202*2*. STCV1; Single-trait Genomic Prediction, MT-CV1; Multi-trait Genomic Prediction (MT) without secondary trait from the kernels in the candidate set, MT-CV2; MT with secondary traits from kernels (MT-CV2), MT-CV3; Multi-trait Genomic Prediction (MT) without secondary trait from the groat in the candidate set, MT-CV4: Multi-trait Genomic Prediction (MT) with secondary trait from the groat in the candidate set, MT-CV5; MT with secondary traits from kernels but without marker information, MT-CV6: Multi-trait Genomic Prediction (MT) with secondary trait from the kernels in the candidate set but phenotypic information available is unreplicated (replication 1 only).

**Supplementary Figure 12**: Prediction accuracy of the Bivariate model (BT) compared to the single trait (ST) for predicting thins percentage and groat percentage using test weight as predictor traits.
